# A thermodynamic and kinetic study of the antioxidant activity of natural hydroanthraquinones†

**DOI:** 10.1039/d0ra04013d

**Published:** 2020-05-27

**Authors:** Quan V. Vo, Nguyen Minh Thong, Trinh Le Huyen, Pham Cam Nam, Nguyen Minh Tam, Nguyen Thi Hoa, Adam Mechler

**Affiliations:** Institute of Research and Development, Duy Tan University Danang 550000 Vietnam vovanquan2@duytan.edu.vn; The University of Danang, Campus in Kon Tum 704 Phan Dinh Phung Kon Tum Vietnam nmthong@kontum.udn.vn; Department of Applied Chemistry, National Chiao Tung University Hsinchu 30010 Taiwan; Department of Chemical Engineering, The University of Danang, University of Science and Technology Danang 550000 Vietnam; Computational Chemistry Research Group, Ton Duc Thang University Ho Chi Minh City Vietnam nguyenminhtam@tdtu.edu.vn; Faculty of Applied Sciences, Ton Duc Thang University Ho Chi Minh City Vietnam; Academic Affairs, The University of Danang-University of Technology and Education 48 Cao Thang Da Nang 550000 Vietnam; Department of Chemistry and Physics, La Trobe University Victoria 3086 Australia

## Abstract

Novel hydroanthraquinones isolated from marine algal-derived endophytic fungus *Talaromyces islandicus* EN-501 exhibited promising antioxidant properties in preliminary studies, raising the prospect of adapting these compounds for therapeutic use in diseases caused by oxidative stress. For medicinal applications it is beneficial to develop a full understanding of the antioxidant activity of these compounds. In this study, the hydroperoxide radical scavenging activity of five natural hydroanthraquinones was evaluated by kinetic and thermodynamic calculations. The results showed that the radical scavenging of these hydroanthraquinones in the gas phase and in lipid solvents was defined by the formal hydrogen transfer mechanism, that for the polar environments was decided by the sequential proton loss electron transfer pathway. The hydroanthraquinones exhibited good hydroperoxide scavenging activity in both polar and non-polar media. The overall rate constant values for the radical scavenging reaction were in the range of 3.42 × 10^1^ to 2.60 × 10^5^ M^−1^ s^−1^ and 3.80 × 10^6^ to 5.87 × 10^7^ M^−1^ s^−1^ in pentyl ethanoate and water solvents, respectively. Thus the activity of 8-hydroxyconiothyrinone B (1) is about 2.6 and 444.6 times higher than that of Trolox in the studied solvents, identifying 8-hydroxyconiothyrinone B as a promising antioxidant.

## Abstract 2

Novel hydroanthraquinones isolated from *Talaromyces islandicus* EN-501 exhibited promising antioxidant properties in preliminary studies, raising the prospect of adapting these compounds for therapeutic use in diseases caused by oxidative stress.

## Graphical abstract

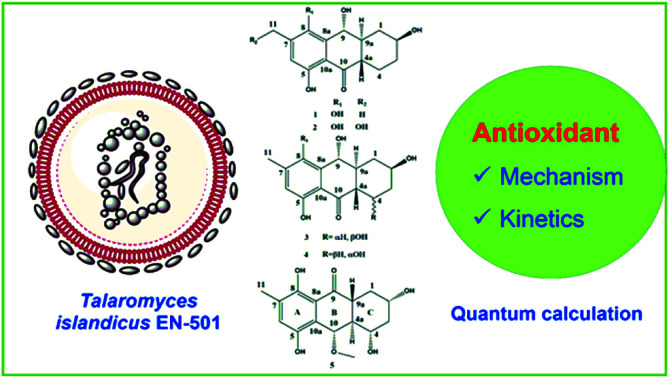

## 1. Introduction

**Fig. 1:**
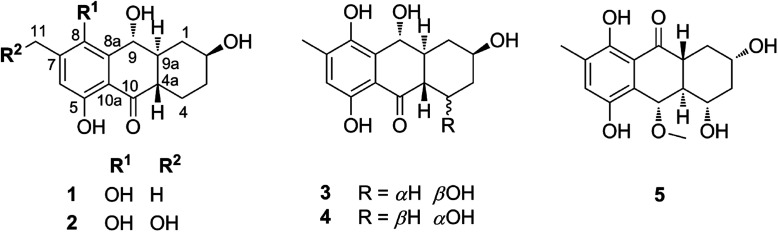
Structures of the five hydroanthraquinones studied here for their antioxidant properties.

In recent trends of drug discovery emphasis has returned to finding bioactive natural products. In nature, marine-derived fungi are known as good sources of hydroanthraquinones whose pharmacological properties have proven beneficial for the treatment of diseases caused by oxidative stress.^1–4^ Recently, five new hydroanthraquinone derivatives from marine algal-derived endophytic fungus *Talaromyces islandicus* EN-501 were isolated and evaluated for biological activity.^3^ The compounds were identified as 8-hydroxyconiothyrinone B (1), 8,11-dihydroxyconiothyrinone B (2), 4-(*R*)-8-dihydroxyconiothyrinone B (3), 4-(*S*)-8-dihydroxyconiothyrinone B (4), and 4-(*S*)-8-dihydroxy-10-*O*-methyldendryol E (5) (Fig. 1). Experimental studies for their antioxidant properties suggested good activity,^3^ warranting a theoretical investigation of the free radical scavenging activity of these compounds that has not been explored thus far.

The relationship between the structural characteristics and the activity of antioxidant compounds can be elucidated based on three main mechanistic pathways of radical scavenging.^5–8^ One of these is the formal hydrogen transfer (FHT) mechanism where the main step is the dissociation of a hydrogen atom from the antioxidant molecule; therefore this mechanism is defined energetically by the bond dissociation enthalpy (BDE). The second common mechanism is the single electron transfer-proton transfer (SET-PT) that is defined by two thermodynamic parameters: ionization energy (IE) (for the electron transfer step) and proton dissociation enthalpy (PDE) (proton transfer from the ionized molecule). The third common mechanism is sequential proton loss electron transfer (SPLET) where the first step is proton dissociation, characterized energetically by proton affinity (PA) and electron transfer enthalpy (ETE) which is the logical next step in the mechanism (Table S1, ESI†).

During the recent years, along with outstanding developments of computing power, the predictive power of computational methods has also increased dramatically, *in silico* study becoming a useful tool for exploring the radical scavenging activity of the potential antioxidant compounds. The computational methods in quantum chemistry provide reasonably accurate information but save time and money compared to experimental methods.^9–13^ Based on a program of evaluating the antioxidant potential of natural products,^10,11,14,15^ this study was carried out to attain three essential goals: (1) establish the most likely mechanism by thermodynamic investigation of the antioxidant activity of hydroanthraquinones through three mechanisms involving SPLET, SETPT, and FHT;^15,16^ (2) approximate radical scavenger activity by performing kinetic evaluation of the HOO˙ scavenging reaction of hydroanthraquinones in the gas phase as well as in physiological environments; and (3) explain the results by analysis of the relationship between environments and molecular structures with the antioxidant activity and oxidation resistance of hydroanthraquinone derivatives.

## 2. Computational methods

Thermochemical properties (*i.e.* BDE, IE and PA) and kinetic parameters (activation energies Δ*G*^≠^ (kcal mol^−1^), tunneling corrections (*κ*) and rate constant (*k*)) in the gas phase as well as physiological environments (water for the aqueous solution and pentyl ethanoate for lipid medium) of the compounds were computed at the M06-2X/6-311++G(d,p) level of theory. This method is proven to be highly accurate for computing both thermodynamic and kinetic parameters with low errors compared to more complex methods (*i.e.* G3(MP2)-RAD) or experimental data.^10,17–20^ The kinetic calculations were computed following the quantum mechanics based test for overall free radical scavenging activity (QM-ORSA) protocol with the solvation model density (SMD) method that has been widely used for evaluating the radical scavenging activity of antioxidants due to low errors compared with experimental data (*k*_calc_/*k*_exp_ ratio = 1–2.9).^9,10,21,22^

The rate constant (*k*) was calculated by using the conventional transition state theory (TST) and 1M standard state as:^23–27^1
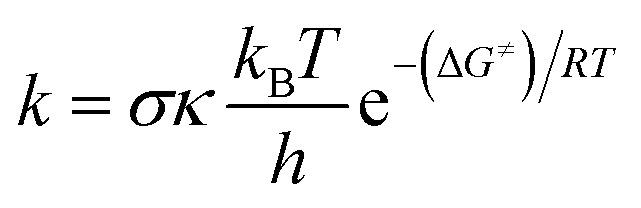
where *σ* the reaction symmetry number,^28,29^*κ* tunneling corrections which were calculated using Eckart barrier,^30^*k*_B_ the Boltzmann constant, *h* the Planck constant, Δ*G*^≠^ Gibbs free energy of activation.

The Marcus theory was used to estimate the reaction barriers of SET reactions.^31–34^ The free energy of reaction Δ*G*^≠^ for the SET pathway was computed following the eqn (2) and (3).2
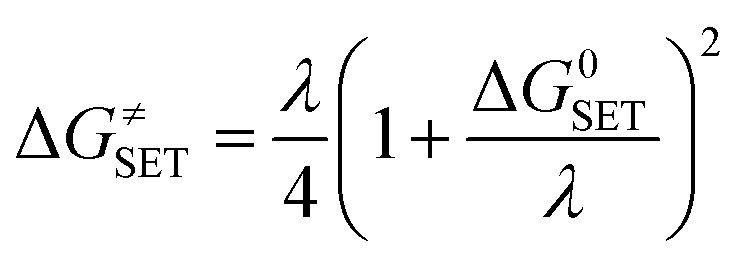

3 *λ* ≈ Δ*E*_SET_ − Δ*G*^0^_SET_

where Δ*G*_SET_ is the Gibbs energy of reaction, Δ*E*_SET_ is the non-adiabatic energy difference between reactants and vertical products for SET.^35,36^ The Collins–Kimball theory in the solvents at 298.15 K was applied to computed the apparent rate constants (*k*_app_) following the eqn (4).^37^ In which, the steady-state Smoluchowski rate constant (*k*_D_) for an irreversible bimolecular diffusion-controlled reaction was calculated following the literature as corroding to eqn (5).^9,38^4
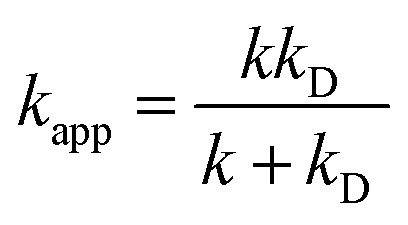

5 *k*_D_ = 4π*R*_AB_*D*_AB_*N*_A_

where *R*_AB_ is the reaction distance, *N*_A_ is the Avogadro constant, and *D*_AB_ = *D*_A_ + *D*_B_ (*D*_AB_ is the mutual diffusion coefficient of the reactants A and B),^37,39^ where *D*_A_ or *D*_B_ is estimated using the Stokes–Einstein formulation (6).^40,41^6
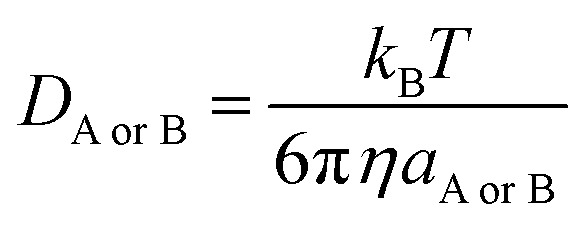


*η* is the viscosity of the solvents (*i.e. η*(pentyl ethanoate) = 8.62 × 10^−4^ Pa s and *η*(H_2_O) = 8.91 × 10^−4^ Pa s) and *a* is the radius of the solute.

The Okuno^42^ and Benson corrections were used to reduced over-penalizing entropy losses in solution.^9,43–45^ For the species that have multiple conformers, all of these were investigated and the conformer with the lowest electronic energy was included in the analysis.^11,46^ All transition states were characterized by the existence of only one single imaginary frequency. Intrinsic coordinate calculations (IRCs) were performed to ensure that each transition state is corrected.^22^ The calculations were performed with the Gaussian 09 suite of programs,^47^ and the Eyringpy code^48,49^ depending on the particular problem. The shape of frontier molecular orbitals (HOMO and SOMO) in transition states that were visualized by using the GaussView 05 software was analyzed to distinguish between HAT and PCET mechanisms.

## 3. Results and discussions

### 3.1. Thermodynamic study

Initially, the antioxidant activity was evaluated by calculating the thermochemical parameters (BDEs, PAs and IEs) that define affinity for the three main mechanisms including FHT, SETPT and SPLET, respectively (Table S1, ESI†).^15,16^ Thus thermochemical characteristics in the gas phase of all of possible X–H (X = C, O) bonds were firstly screened by using DFT calculation at the M06-2X/6-31G level (Table S2, ESI†); the X–H (X = C, O) bonds with the lowest BDEs or PAs were then computed at the higher level M06-2X/6-311++G(d,p). The results are shown in Table 1.

**Table 1:** The calculated BDEs, PAs and IEs (kcal mol^−1^) in the gas phase of the studied compounds^a^

Comp.	X–H	BDE	PA	IEs
1	C9–H	77.6		176.1
	O8–H	76.8	319.9	
2	C9–H	76.2		172.7
	O8–H	79.0	313.8	
3	C9–H	79.5		180.1
	O8–H	77.6	318.4	
4	C9–H	76.4		180.3
	O8–H	77.1	318.4	
5	C10–H	84.8		177.3
	O5–H	88.6	331.6	

a The atom numbers (C9, O5, O8, C10) are showed in Fig. 1.

As shown in Table 1, the BDE(O–H) values are in the range of 76.8 to 88.6 kcal mol^−1^, whereas those for C–H bonds are 76.2–84.8 kcal mol^−1^. In the antioxidant activity of 5 following the FHT mechanism the C10–H and O5–H bonds were dominant, while for compound 1, 2, 3 and 4 the lowest BDEs were observed at the O8–H bond and C9–H bond (at about 76–79 kcal mol^−1^). Thus in gas phase these compounds appear to be potent radical scavengers according to the FHT pathway.

The computed PA and IE values are in the gas phase were in the range of 313.8 to 331.8 kcal mol^−1^ and 172.7 to 180.3 kcal mol^−1^, respectively. It was found that compound 2 has the lowest PA and IE values (PA = 313.8 and IE = 172.7 kcal mol^−1^), thus the radical scavenging of this compound may be followed the SETPT and SPLET pathways in the gas phase.

To investigate the favored antioxidant mechanism of the studied compounds, the free energy (Δ*G*^o^) of the first step for the HOO˙ scavenging of the hydroanthraquinones following each mechanism were calculated in vacuum and shown in Table S3, ESI.† The results show that only FHT mechanism yields negative Δ*G*^o^, whereas the reactions following the SETPT and SPLET mechanisms are not spontaneous. Hence, the FHT pathway is suggested to be the main antiradical mechanism for the neutral hydroanthraquinones in the gas phase.

### 3.2. Kinetic study

#### 3.2.1. The HOO˙ radical scavenging of hydroanthraquinones in the gas phase

**Fig. 2:**
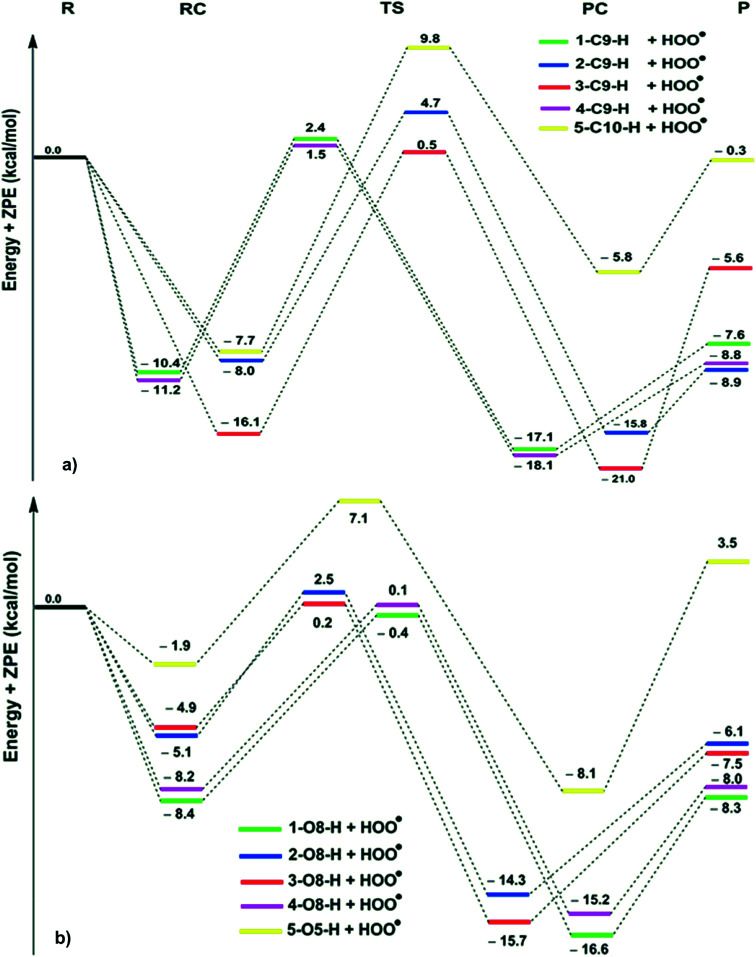
PES for the reactions of the hydroanthraquinones with HOO˙ in the gas phase ((a) C–H; (b) O–H; R: reagent, RC: pre-complex; TS: transition state; PC: post-complex; P: products).

**Fig. 3:**
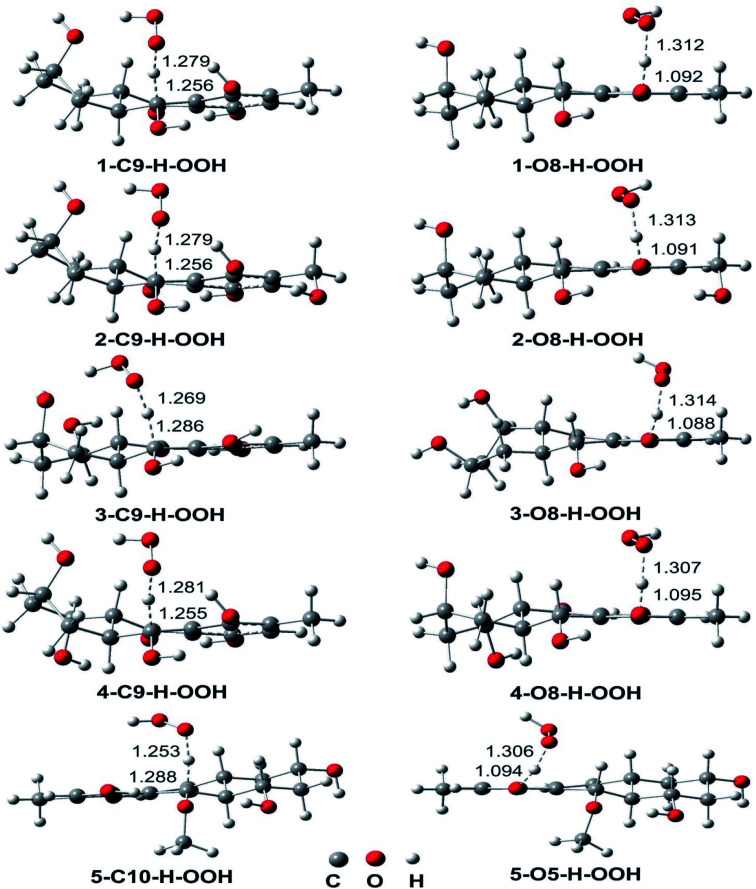
Optimized geometries of TS_S_ between the studied compounds and HOO˙ radical in the gas phase following the FHT mechanism.

The obtained results in the thermodynamic section showed that the FHT is the key mechanism for the HOO˙ scavenging of the hydroanthraquinones. Thus in this section, the kinetic study was focused on the H-abstraction at the C–H and O–H. The kinetic parameters (calculated activation energies Δ*G*^≠^ (kcal mol^−1^), tunneling corrections (*κ*) and *k*_Eck_ (M^−1^ s^−1^) at 298.15 K in the gas phase), the potential energy surfaces (PES) and optimized TS structures are presented in Table 2, Fig. 2 and 3, respectively.

**Table 2:** Calculated Δ*G*^≠^ (kcal mol^−1^), *κ* and *k*_Eck_ (M^−1^ s^−1^) for the HOO˙ scavenging of the hydroanthraquinones in the gas phase

Reactions	Δ*G*^≠^	*κ*	*k*_Eck_
1–O8–H + HOO˙	8.5	20.9	7.23 × 10^7^
1–C9–H + HOO˙	12.2	68.4	5.18 × 10^5^
2–O8–H + HOO˙	11.0	19.4	1.02 × 10^6^
2–C9–H + HOO˙	14.4	68.2	1.14 × 10^4^
3–O8–H + HOO˙	9.3	14.1	1.33 × 10^7^
3–C9–H + HOO˙	10.4	65.7	1.07 × 10^7^
4–O8–H + HOO˙	9.8	23.6	1.02 × 10^7^
4–C9–H + HOO˙	11.4	63.6	1.69 × 10^6^
5–O5–H + HOO˙	15.7	14.4	2.89 × 10^2^
5–C10–H + HOO˙	16.2	160.0	1.39 × 10^3^
Trolox + HOO˙	9.7	36.7	1.87 × 10^7^

The reaction proceeds *via* reaction complexes (RC) that are energetically more stable than the reactants: about 7.7–16.1 kcal mol^−1^ for the H-abstraction of the C–H bonds and 1.9–8.4 kcal mol^−1^ for the H-abstraction of the O–H bonds. After that, the reactions can proceed to transition states (TS) from the RC by FHT process; TSs have higher energy barriers than RC: around 12.0–17.0 kcal mol^−1^ in the case of (C–H) and 5.1–9.0 kcal mol^−1^ at (O–H). The energy barrier of the reaction path *via* C–H positions is higher than that in case of O–H positions at an average by 6.9 kcal mol^−1^. The lowest TS energy (−0.4 kcal mol^−1^) was observed at the HOO˙ antiradical of the 1–O8–H bond that correlates with the lowest calculated BDE value of O–H bonds (BDE(1–O8–H) = 76.8 kcal mol^−1^). Comparing the energy barriers of the transition states of the reaction pathways at both C–H and O–H positions of hydroanthraquinones, one can make a conclusion that the reaction at the O–H bond is energetically preferred over the C–H bond.

As shown in Table 2, the rate constants for the hydroanthraquinones + HOO˙ reactions in the gas phase are in the range of 2.89 × 10^2^ to 7.23 × 10^7^ M^−1^ s^−1^, while the Δ*G*^≠^ values for these processes are from 8.5 to 16.2 kcal mol^−1^. The tunneling corrections (*κ*) for the HOO˙ radical scavenging of the O–H bonds (14.1–23.6) are lower than those of the C–H bonds (63.6–160.0). Based on the calculated data, among the studied compounds the HOO˙ scavenging activity of 1 is the fastest with *k*_Eck_ = 7.23 × 10^7^ M^−1^ s^−1^; it is nearly four times higher than that of Trolox (*k*_Eck_ = 1.87 × 10^7^ M^−1^ s^−1^). The compounds 3 and 4 also exhibit an excellent hydroperoxyl radical scavenging (*k*_Eck_ = ∼10^7^ M^−1^ s^−1^), whereas that for compound 5 is the lowest with *k*_Eck_ = ∼10^3^ M^−1^ s^−1^. This result is in good agreement with the obtained BDE values in the thermodynamic evaluation. Thus compounds 1, 3 and 4 are promising scavengers in the gas phase.

The effect of the explicit presence of a solvent, *i.e.* water, molecule on the radical scavenging of the most active antioxidant (compound 1) was also investigated given the potential influence of hydrogen bonding on the proton dissociation process (Table S4 and Fig. S1, ESI†). The presence of the H_2_O molecule in the reaction can have a substantial effect on the rate constant of the HOO˙ radical scavenging reaction of 1. For examples, the 1–O8–H(H_2_O) + ˙OOH and 1–O8–H(H_2_O) + ˙OOH(H_2_O) reactions in the gas phase have rate constants *k*_Eck_ = 5.06 × 10^5^ and 2.89 × 10^6^ M^−1^ s^−1^, respectively, compared with *k*_Eck_ = 7.23 × 10^7^ M^−1^ s^−1^ for the 1–O8–H + ˙OOH reaction. This is contrary to the intuition that H-bonding to water should promote bond dissociation, and is the result of explicit inclusion of a water molecule. In an environment where there is competition for hydrogen bonding this effect might be less pronounced.

**Fig. 4:**
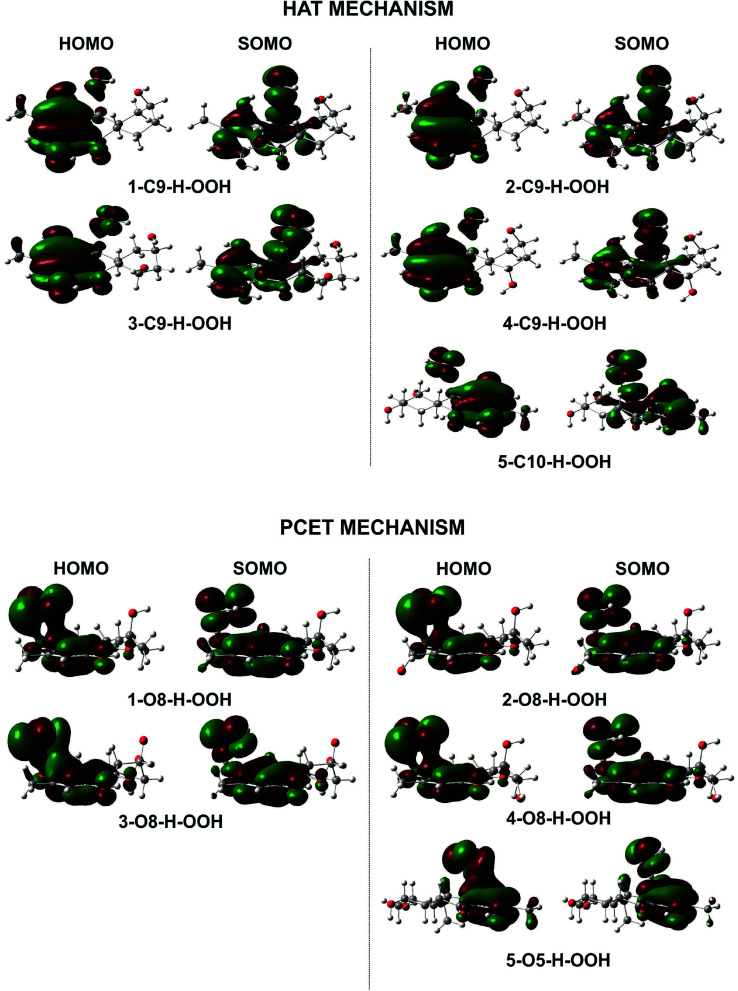
HOMO and SOMO density surfaces of transition states for the studied compounds reaction with HOO˙ radical.

To gain further into the mechanism of the H-abstraction of the O–H and C–H bonds, frontier molecular orbital (FMO) analysis of the transition states was performed and the results are shown in Fig. 4.^50,51^ There is an overlap in the highest occupied molecular orbital (HOMO) density surfaces between delocalized π-orbitals of the rings and a lone pair on the central peroxyl oxygen of the hydroperoxyl radical in case of the TSs that were formed by H-abstraction from the O8(5)H bond. This overlap allows electron transfer between the two in the TS structures. Moreover, the singly-occupied molecular orbitals (SOMO) of transition states involve p type orbitals, which are orthogonal to the transition vector. That suggests that the reaction between the studied compounds and HOO˙ in position O5, and O8 occurs *via* the proton coupled electron transfer (PCET) mechanism.^10,52^ On the other hand a significant atomic orbital density oriented along the C⋯H⋯O transition vector is observed in the SOMO density surfaces of the TSs formed by H-abstraction of the C9(10)–H bond. It means that the single entity (H˙) is transferred along the line connecting the C9(10) and O centers, which corresponds to hydrogen atom transfer (HAT) mechanism.^52,53^ Thus the FMO analysis shows that the HOO˙ radical scavenging of C9(10)–H bond follows the HAT mechanism, whereas the PCET pathway is favored at the O8(5)–H bonds. This may explain the higher rare constants for the H-abstraction from the O–H bonds compared to the C–H bonds despite of the lower BDE values at the C9–H (compound 2 and 4) and C10–H (compound 5) bonds compared to the O–H bonds of these compounds.

#### 3.2.2. HOO˙ scavenging of hydroanthraquinones in physiological environments

##### Acid–base equilibria

Previous studied showed that the antioxidant activity should be evaluated in physiological environments that provides more accurate data that correlates well with experimental results.^9,10^ Thus in this section the antioxidant activity of the hydroanthraquinones was investigated against HOO˙ radical in aqueous solution (water, pH = 7.4) and lipid environment (pentyl ethanoate) that mimic the polar and nonpolar environments in the human body.^9–11,15,46^ To determine the structure of the studied compounds in the aqueous solution, knowing protonation state is important. The p*K*_a_ (negative logarithm of the acid dissociation constant) values and the molar fractions of five hydroanthraquinones were computed by using the model reaction (7) and eqn (8) following the literature^9,54–56^ and are shown in Table 3.

7 HA + Ref^−^ → A^−^ + HRef

8 p*K*_a_ = Δ*G*_s_/*RT* ln(10) + p*K*_a_(HRef)

where Δ*G*_s_ is the reaction free energy in solution and the HRef is phenol with the experimental p*K*_a_(O–H) = 10.09.^57^

**Table 3:** Calculated p*K*_a_ and *f* at pH = 7.4

Comp.	OH position	p*K*_a_	*f*_protonated_(HA)	*f*_deprotonated_(A^−^)
1	O8–H	8.57	0.937	0.063
2	O8–H	8.24	0.874	0.126
3	O8–H	8.50	0.926	0.074
4	O8–H	8.50	0.926	0.074
5	O5–H	8.69	0.951	0.049

As can be seen from Table 3, the calculated p*K*_a_ values are from 8.24 to 8.69. The *f*_protonated_(HA) values are in the range of 0.874 to 0.951, whereas those for the *f*_deprotonated_(A^−^) are in the range of 0.049 to 0.126. Thus in the water solvent (pH = 7.4), the hydroanthraquinones exist in both anionic and neutral states and these states were both included in the further study.

##### Kinetic study

As shown in thermodynamic section, the HOO˙ antiradical activity of the hydroanthraquinones in the gas phase was decided by the FHT mechanism, which is a good indication for the dominant HOO˙ radical scavenging pathway in the nonpolar medium. In the polar environment the HOO˙ radical scavenging is, however, affected by interaction with water that leads to concurrent pathways following the FHT mechanism (for the neutral states) and SET mechanism (for the anionic states).^15^ Therefore, the overall rate constants (*k*_overall_) *i.e.* the total of all rate constants of the studied mechanistic pathways^9^ were calculated according to the eqn (9) and (10). The branching ratios (*Γ*) that characterize the contribution of each reactions mechanism or pathways in the overall rate constant^9,10^ were computed following the eqn (11). The obtained results are shown in Table 4.

**Table 4:** The calculated Δ*G*^≠^ (in kcal mol^−1^), *k*_app_ (M^−1^ s^−1^) and *Γ* (%) of the studied compounds + HOO˙ reaction in water and pentyl ethanoate solvents

Comp.	Pentyl ethanoate						Water				
	Mechanism			Δ*G*^≠^	*k*_app_	*Γ*	Δ*G*^≠^	*k*_app_	f	*k*_f_ a	*Γ*
1	SET						5.1	9.30 × 10^8^	0.063	5.86 × 10^7^	99.7
	FHT	O8		12.2	2.60 × 10^5^	100.0	13.4	1.70 × 10^5^	0.937	1.59 × 10^5^	0.3
	*k*_overall_				**2.60 × 10** ^**5**^					**5.87 × 10** ^**7**^	
2	SET						7.2	3.00 × 10^7^	0.126	3.78 × 10^6^	99.4
	FHT	O8		14.9	1.90 × 10^3^	100.0	14.5	2.50 × 10^4^	0.874	2.19 × 10^4^	0.6
	*k*_overall_				**1.90 × 10** ^**3**^					**3.80 × 10** ^**6**^	
3	SET						5.4	6.60 × 10^8^	0.074	4.88 × 10^7^	100
	FHT	O8		13.5	4.80 × 10^4^	83.0	14.1	4.80 × 10^4^	0.926	4.44 × 10^4^	0.1
		C9		15.1	7.00 × 10^3^	17.0	15.5	7.00 × 10^3^		6.48 × 10^4^	0.0
	*k*_overall_				**2.89 × 10** ^**4**^					**4.89 × 10** ^**7**^	
4	SET						5.6	4.70 × 10^8^	0.074	3.48 × 10^7^	99.8
	FHT	O8		13.4	3.90 × 10^4^	99.2	14.3	6.60 × 10^4^	0.926	6.11 × 10^4^	0.2
		C9		16.7	3.10 × 10^2^	0.8	16.4	7.60 × 10^2^		7.04 × 10^2^	0.0
	*k*_overall_				**3.93 × 10** ^**4**^					**3.48 × 10** ^**7**^	
5	SET						5.2	9.20 × 10^8^	0.049	4.51 × 10^7^	100.0
	FHT	O5		18.1	2.70 × 10^1^	100.0	17.3	4.55 × 10^2^	0.951	4.32 × 10^2^	0.0
		C10		19.5	7.20	26.7	18.6	2.40 × 10^1^		2.28 × 10^1^	0.0
	*k*_overall_				**3.42 × 10** ^**1**^					**4.51 × 10** ^**7**^	
Trolox			12.6		**1.00 × 10** ^**5**^		11.7			**1.30 × 10** ^**5**^	

a *k*_f_ = *f* × *k*_app_.

Rate constant in lipid medium:

9 *k*_overall_ = ∑*k*^FHT^_app_(X–H)

where the X–H bonds are O8–H and C9–H bonds for compound 3 and 4; O5–H and C10–H bonds for compound 5 and O8–H bond for compounds O8–H and 2.

Rate constant in aqueous medium:

10 *k*_overall_ = *f*_A^−^_*k*^SET^_app_(A^−^) + *f*_HA_*k*^FHT^_app_(HA) = *k*^SET^_f_(A^−^) + *k*^FHT^_f_(HA)

11
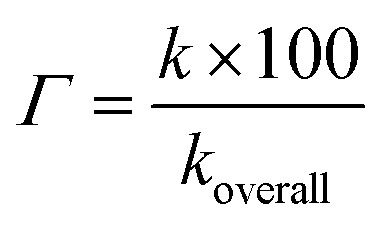


As shown in Table 4, the HOO˙ radical scavenging activity of the hydroanthraquinones is more than 200 times higher in water than in pentyl ethanoate solvent. The *k*_overall_ values in the nonpolar environment are defined by the FHT pathway of the O–H bonds (*Γ* = 83−100%) and are in the range of 3.42 × 10^1^ to 2.60 × 10^5^ M^−1^ s^−1^, whereas those for the polar solvent is decided by the SET mechanism (*Γ* = 99.4–100.0%, *k*_overall_ = 3.80 × 10^6^ to 5.87 × 10^7^ M^−1^ s^−1^). This result suggests that the SET mechanism plays a deciding role in the antioxidant activity of the hydroanthraquinones in polar environments. The highest overall rate constant was observed at compound 1 with *k*_overall_ = 2.60 × 10^5^ M^−1^ s^−1^ and 5.87 × 10^7^ M^−1^ s^−1^ in polar and non-polar media, respectively. The compounds 3 and 4 also exhibit excellent HOO˙ radical scavenging with *k*_overall_ = 2.89 × 10^4^ and 3.93 × 10^4^ M^−1^ s^−1^ in lipid medium and 4.89 × 10^7^ and 3.48 × 10^7^ M^−1^ s^−1^ in the aqueous solution, respectively. Compound 5 exhibits the lowest radical scavenging in lipid medium (*k*_overall_ = 3.42 × 10^1^ M^−1^ s^−1^), however this value in the polar environment is the second highest with *k*_overall_ = 4.51 × 10^7^ M^−1^ s^−1^. Comparing the obtained results with Trolox (*k*_overall_ = 1.00 × 10^5^ and 1.30 × 10^5^ M^−1^ s^−1^ in pentyl ethanoate and water, respectively, Table 4) the studied compounds exhibit higher HOO˙ radical scavenging than the reference compound Trolox in the aqueous solution. The HOO˙ radical scavenging of 1 is about 2.6 and 444.6 times higher than that of Trolox in the nonpolar and polar environments, respectively. Hence, 1 is the most potential antioxidant in physiological environments. This is in good agreement with the experimental data of the DPPH and ABTS testing.^58^

## 4. Conclusions

The hydroperoxide radical scavenging activity of five natural hydroanthraquinones was evaluated by thermodynamic and kinetic calculations. The results showed that the formal hydrogen transfer pathway is the main mechanism for the antiradical activity of these hydroanthraquinones in nonpolar environments. It was found that the H-abstraction of O8–H bond plays a deciding role in the antioxidant activity of the studied compounds. However, the SET mechanism is favored in polar environment. It is important to notice that most of the studied compounds exhibit excellent HOO˙ scavenging activity in both polar and non-polar environments. In particular the HOO˙ radical scavenging of 1 is about 2.6 and 444.6 times higher than that of Trolox in the studied solvents. Hence, compound 1 is a potent antioxidant in physiological environments.

## Conflicts of interest

There are no conflicts to declare.

## Supplementary Material

RA-010-D0RA04013D-s001
