# Supplementary material for: A thermodynamic and kinetic study of the antioxidant activity of natural hydroanthraquinones
Source: RSC Adv. 2020 May 27;10(34):20089–97. doi: 10.1039/d0ra04013d (PMC9054120; doi:10.1039/d0ra04013d)
Supplement: RA-010-D0RA04013D-s001 [file RA-010-D0RA04013D-s001.pdf]

Electronic Supplementary Material (ESI) for RSC Advances.
This journal is © The Royal Society of Chemistry 2020

Supporting Information (SI)

A thermodynamic and kinetic study of the antioxidant activity of natural hydroanthraquinones

Quan V. Vo[1*], Nguyen Minh Thong[2*], Trinh Le Huyen[3], Pham Cam Nam[4], Nguyen Minh Tam[5,6*] Nguyen Thi Hoa[7] and Adam Mechler[8]

[1]Institute of Research and Development, Duy Tan University, Danang 550000, Vietnam

[2]The University of Danang, Campus in Kon Tum, 704 Phan Dinh Phung, Kon Tum, Vietnam

[3]Department of Applied Chemistry, National Chiao Tung University, Hsinchu 30010, Taiwan

[4]Department of Chemical Engineering, The University of Danang - University of Science and Technology, Danang 550000, Vietnam.

[5]Computational Chemistry Research Group, Ton Duc Thang University, Ho Chi Minh City, Vietnam.

[6]Faculty of Applied Sciences, Ton Duc Thang University, Ho Chi Minh City, Vietnam

[7]Academic Affairs, The University of Danang - University of Technology and Education, Da Nang 550000, Vietnam.

[8]Department of Chemistry and Physics, La Trobe University, Victoria 3086, Australia

*Corresponding author: vovanquan2@duytan.edu.vn, nmthong@kontum.udn.vn; nguyen minhtam@tdtu.edu.vn

Table of Contents

Table S1. The method to compute the thermochemical properties S2

Table S2. BDE and PA values of the X-H (X = O, C) bonds of the studied compounds in the gas phase at the M06-2X/6-31g level of theory S3

Table S3. The Calculated Free Energy (ΔG°, in kcal/mol at 298.15 k) of the Reaction Between the Selected Compounds with $HOO^{\bullet}$ Radical via the Formal Hydrogen Transfer (FHT), Sequential Proton (SA) and SET Processes in the Gas Phase. S5

Table S4. Calculated $\Delta G^{\neq}$ (kcal/mol), κ and k_{Eck} ($M^{-1}s^{-1}$) for the $HOO^{\bullet}$ scavenging of the hydroanthraquinones in the presence of one H_2O molecule in the gas phase. S6

Figure S1. Optimized geometries of TSs between the 1-O8-H and a HOO• radical in the presence of one H_2O molecule in the gas phase S6

Table S5: The cartesian coordinates and energies of TS of the reaction between selected compounds with $HOO^{\bullet}$ at the M06-2x/6-311++G(d,p) calculating method following the FHT mechanism S7

Table S1. The method to compute the thermochemical properties

In order to determine the mechanistic pathway of the radical scavenging process, thermochemical properties were used, by assessing the energetics of the determining step of each pathway. The literature recognizes three common mechanisms of antioxidant activity.[1-5] In the formal hydrogen transfer (FHT) mechanism, the first step is the homolytic bond breakage in an appropriate moiety to yield a hydrogen radical, which then reacts with the free radical species; here the bond dissociation energy of the R–H moiety determines the enthalpy of the first step.

$$R\text{–}H \rightarrow R^{\bullet} + H^{\bullet} \quad (BDE(R\text{–}H)) \qquad (1)$$

In the "Single electron transfer followed by proton transfer" (SETPT) mechanism the first step is electron loss to form a radical cation, characterized by the ionization energy, followed by a deprotonation step that is described with the proton dissociation energy.

$$R\text{–}H \rightarrow RH^{+\bullet} + e^{-} \quad (IE) \qquad (2)$$

$$RH^{+\bullet} \rightarrow R^{\bullet} + H^{+} \quad (PDE) \qquad (3)$$

The third mechanism, "Sequential proton loss electron transfer" (SPLET), starts with the dissociation of the acidic moiety, which can be characterized by the proton affinity; this is followed by an electron transfer to the free radical, at a cost of the electron transfer energy

$$R\text{–}H \rightarrow R^{-} + H^{+} \quad (PA) \qquad (4)$$

$$R^{-} \rightarrow R^{\bullet} + e^{-} \quad (ETE) \qquad (5)$$

Thus the reaction enthalpies of the individual steps in the above described mechanisms of antioxidant activity in gas phase (at 298.15 K and 1 atm) are calculated as follows:[1,6,7]

$$BDE = H(R^{\bullet}) + H(H^{\bullet}) - H(R\text{–}H) \qquad (6)$$

$$IE = H(RH^{\bullet+}) + H(e^{-}) - H(R\text{–}H) \qquad (7)$$

$$PDE = H(R^{\bullet}) + H(H^{+}) - H(R^{\bullet+}) \qquad (8)$$

$$PA = H(R^{-}) + H(H^{+}) - H(R\text{–}H) \qquad (9)$$

$$ETE = H(R^{\bullet}) + H(e^{-}) - H(R^{-}) \qquad (10)$$

In the gas phase, the enthalpy of hydrogen atom was calculated at the same method (the M06-2x/6-311++G(d,p) level of theory. The calculated enthalpies of the electron (e^{-}) and proton (H^{+}) were taken from the

literature[1,8-10].

Table S2. BDE and PA values of the X-H (X = O, C) bonds of the studied compounds in the gas phase at the M06-2X/6-31g level of theory

Compounds	Position	BDEs (kcal/mol)	PAs (kcal/mol)
**1**			
	C1-H	96.4	391.5
	C2-H	92.5	402.6
	C3-H	93.9	390.2
	C4-H	94.1	391.3
	C4a-H	79.9	342.1
	C6-H	115.8	391.7
	C9-H	**72.3**	354.8
	C9a-H	94.0	384.6
	O2-H	93.9	371.3
	O5-H	99.4	350.1
	O8-H	**70.2**	**322.1**
	O9-H	88.1	367.3
**2**			
	C1-H	96.3	391.2
	C2-H	91.8	401.7
	C3-H	93.5	390.8
	C4-H	94.1	391.2
	C4a-H	79.2	340.1
	C6-H	114.9	385.1
	C9-H	**69.9**	356.2
	C9a-H	91.8	385.1
	C11-H	79.7	348.2
	O2-H	93.9	371.8
	O5-H	99.1	347.2
	O8-H	**75.2**	**318.1**
	O9-H	99.8	363.2
	O11-H	93.9	371.0
**3**			
	C1-H	96.1	386.7
	C2-H	93.3	396.1
	C3-H	97.1	380.2
	C4-H	96.4	403.6
	C4a-H	83.1	327.7
	C6-H	116.6	387.1
	C9-H	**73.7**	353.2
	C9a-H	91.4	379.1
	O2-H	94.3	363.5
	O4-H	93.4	348.3

	O5-H	93.1	347.6
	O8-H	**71.2**	**321.1**
	O9-H	84.6	363.1
**4**			
	C1-H	96.5	390.3
	C2-H	93.1	389.1
	C3-H	96.1	371.8
	C4-H	87.3	388.4
	C4a-H	81.2	334.8
	C6-H	116.1	386.1
	C9-H	**71.1**	345.8
	C9a-H	94.4	365.4
	O2-H	94.4	371.0
	O4-H	96.9	347.2
	O5-H	91.7	343.5
	O8-H	**71.3**	**321.2**
	O9-H	88.5	366.2
**5**			
	C1-H	97.8	384.3
	C2-H	89.1	389.8
	C3-H	97.9	388.1
	C4-H	89.8	385.0
	C4a-H	95.2	367.9
	C6-H	115.1	385.7
	C9a-H	83.7	334.9
	C10-H	**81.1**	348.2
	O2-H	94.3	361.1
	O4-H	97.1	354.0
	O5-H	**84.5**	**334.1**
	O8-H	89.3	341.6

Table S3. The Calculated Free Energy (ΔG^o, in kcal/mol at 298.15 k) of the Reaction Between the Selected Compounds with $HOO^{\bullet}$ Radical via the Formal Hydrogen Transfer (FHT), Sequential Proton (SA) and SET Processes in the Gas Phase.

The Formal Hydrogen Transfer (FHT) mechanism: $R\text{-}H + HOO^{\bullet} \rightarrow R^{\bullet} + HOOH$

The Sequential Proton (SA) mechanism: $R\text{-}H + HOO^{\bullet} \rightarrow R^{-} + HOOH^{+\bullet}$

The Single Electron Transfer (SET) mechanism: $R\text{-}H + HOO^{\bullet} \rightarrow RH^{\bullet+} + HOO^{-}$

compounds	Position	SA		SET		FHT	
		ΔH	ΔG	ΔH	ΔG	ΔH	ΔG
**1**	O8-H	197.1	198.1	154.2	153.9	-8.5	-8.6
	C9-H					-7.7	-7.6
**2**	O8-H	191.5	192.0	151.7	150.8	-6.2	-6.8
	C9-H					-9.0	-9.6
**3**	O8-H	196.6	196.7	157.5	157.1	-7.7	-7.9
	C9-H					-5.7	-6.5
**4**	O8-H	196.4	196.8	156.9	156.4	-8.2	-8.7
	C9-H					-8.9	-9.0
**5**	O5-H	199.8	201.3	154.2	153.9	3.3	2.0
	C10-H					-0.5	-1.4

Table S4. Calculated ΔG≠ (kcal/mol), κ and k_{Eck} ($M^{-1}s^{-1}$) for the HOO• scavenging of the hydroanthraquinones in the presence of one H_2O molecule in the gas phase.

REACTIONS	ΔG≠	κ	k_{Eck}
**1-O8-H + •OOH**	8.5	20.9	7.23×10^{7}
**1-O8-H-H_2O + •OOH**	11.3	14.6	5.06×10^{5}
**1-O8-H + •OOH-H_2O**	10.2	14.3	2.89×10^{6}

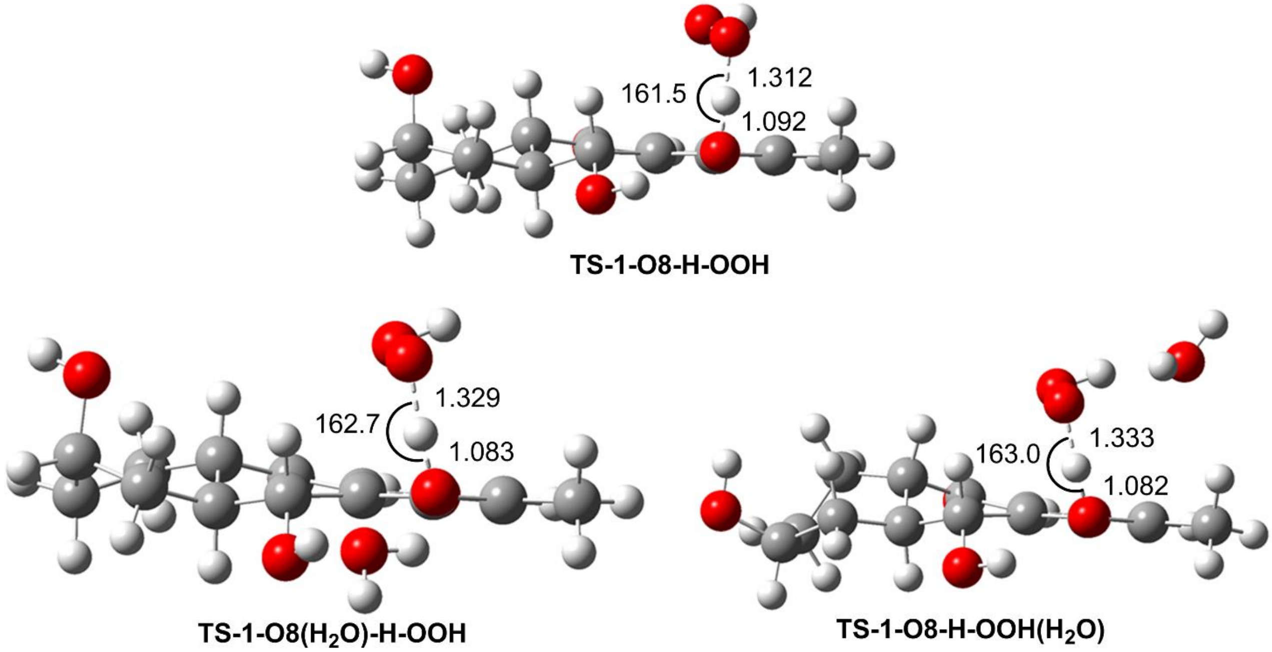

Figure S1. Optimized geometries of TSs between the **1**-O8-H and a HOO• radical in the presence of one H_2O molecule in the gas phase

Table S5: The cartesian coordinates and energies of TS of the reaction between selected compounds with HOO• at the M06-2x/6-311++G(d,p) calculating method following the FHT mechanism

**Name**	**1-O8-H-OOH**
Cartesian Coordinates	Frequency and Energy
C 3.23384100 0.36574800 -0.45144700<br>C 2.16065900 -0.56546300 -0.52707400<br>C 0.82457800 -0.14021200 -0.37284200<br>C 0.56108400 1.20483700 -0.09843900<br>C 1.63605900 2.13020100 -0.03271800<br>C 2.95782500 1.69340600 -0.21500100<br>C -0.29402400 -1.16363500 -0.44634400<br>C -0.80988600 1.66898200 0.19413600<br>C -1.92076900 0.65070600 0.26650600<br>C -1.66617200 -0.53826600 -0.66213500<br>C -2.75298600 -1.62069800 -0.50239800<br>H -2.42886000 -2.36623600 0.22920100<br>C -4.09851400 -1.06888800 -0.03553100<br>C -4.31329000 0.33599900 -0.58623000<br>C -3.28418600 1.31859400 -0.00594000<br>H 3.74826800 2.43214400 -0.15228600<br>H -4.89809400 -1.73762200 -0.37336400<br>H -4.22634000 0.28657300 -1.67668100<br>H -3.65380000 1.75198600 0.92376900<br>H -3.14464600 2.15359700 -0.69556500<br>H -5.32699300 0.68669100 -0.37017300<br>H -2.87488700 -2.14173900 -1.45311800<br>H -0.29472300 -1.69182100 0.52240900<br>C 4.63321300 -0.14762600 -0.61408100<br>H 5.35962400 0.65906900 -0.52471700<br>H 4.85062100 -0.91172500 0.13833100<br>O 2.44530900 -1.85280600 -0.71884600<br>H 2.67293100 -2.30329700 0.24992100<br>O -0.12725600 -2.08047700 -1.50686100<br>H 0.75701700 -2.45973500 -1.43696000<br>O -4.07218000 -1.05278900 1.39369200<br>H -4.91984100 -0.73344400 1.71497900<br>O 1.44624800 3.41934700 0.21082000<br>H 0.47688300 3.55994200 0.32970000<br>O -1.03415800 2.85593800 0.40682600<br>H -1.90949900 0.27264300 1.29956800<br>H -1.67426600 -0.16864200 -1.69619600<br>O 2.11937200 -1.40766700 2.02741300<br>H 2.78632000 -0.73457500 2.23808200<br>O 2.81631800 -2.46457200 1.54405400<br>H 4.74647500 -0.63169900 -1.58645500	Zero-point correction= 0.328802 (Hartree/Particle)<br>Thermal correction to Energy= 0.349322<br>Thermal correction to Enthalpy= 0.350266<br>Thermal correction to Gibbs Free Energy= 0.278822<br>Sum of electronic and zero-point Energies= -1109.030572<br>Sum of electronic and thermal Energies= -1109.010051<br>Sum of electronic and thermal Enthalpies= -1109.009107<br>Sum of electronic and thermal Free Energies= -1109.080551
**Name**	**1-O8-H-OOH (water)**
Cartesian Coordinates	Frequency and Energy
C 3.22232900 0.36841900 -0.44986300<br>C 2.14415200 -0.55542300 -0.53486100	Zero-point correction= 0.327265 (Hartree/Particle)

C 0.80737800 -0.12808600 -0.39228100<br>C 0.54608400 1.21712800 -0.11723600<br>C 1.62823200 2.13415400 -0.05124500<br>C 2.94697900 1.69858300 -0.22452400<br>C -0.31268000 -1.14891600 -0.46308700<br>C -0.82308500 1.67440600 0.18022400<br>C -1.92529100 0.65195500 0.27774600<br>C -1.68876500 -0.53042200 -0.66607200<br>C -2.77121500 -1.61697800 -0.49701100<br>H -2.43043500 -2.36977600 0.22065900<br>C -4.12023200 -1.08189600 -0.02124600<br>C -4.33766800 0.33503000 -0.52762800<br>C -3.30475800 1.30684300 0.06050700<br>H 3.74551300 2.42791900 -0.15231100<br>H -4.91651700 -1.73767100 -0.38468700<br>H -4.26020700 0.31066900 -1.61897800<br>H -3.66114100 1.70412700 1.01191000<br>H -3.19608800 2.15869300 -0.61350400<br>H -5.34792800 0.67403000 -0.28429600<br>H -2.91404600 -2.12715800 -1.45097300<br>H -0.30688600 -1.68821500 0.49701100<br>C 4.62211000 -0.14544600 -0.56758000<br>H 5.34129100 0.66682300 -0.47339400<br>H 4.81387200 -0.88620400 0.21544400<br>O 2.41998500 -1.84942500 -0.74907100<br>H 2.62038500 -2.32076800 0.20179200<br>O -0.13184300 -2.07209000 -1.53034200<br>H 0.75192500 -2.45411300 -1.43367000<br>O -4.12396000 -1.11420100 1.41565800<br>H -4.95090600 -0.72189700 1.72006800<br>O 1.44068600 3.43577300 0.19028000<br>H 0.47422900 3.58379800 0.31043200<br>O -1.05114000 2.86564100 0.39237800<br>H -1.85557600 0.27265100 1.30824800<br>H -1.71854400 -0.14529600 -1.69312200<br>O 2.43877500 -1.33403200 2.02060700<br>H 3.28288200 -0.85310800 2.09998700<br>O 2.75228200 -2.54693900 1.51181600<br>H 4.76849300 -0.64843200 -1.52629400	Thermal correction to Energy= 0.347846<br>Thermal correction to Enthalpy= 0.348790<br>Thermal correction to Gibbs Free Energy= 0.277711<br>Sum of electronic and zero-point Energies= -<br>1109.066563<br>Sum of electronic and thermal Energies= -<br>1109.045982<br>Sum of electronic and thermal Enthalpies= -<br>1109.045038<br>Sum of electronic and thermal Free Energies= -<br>1109.116117
**Name**	**1-O8-H-OOH (pentyl ethanoate)**
Cartesian Coordinates	Frequency and Energy
C 3.20501600 0.38929300 -0.47825200<br>C 2.13889800 -0.55292200 -0.52580300<br>C 0.79915700 -0.13858000 -0.36886000<br>C 0.52796100 1.20556500 -0.09998300<br>C 1.59776800 2.13917600 -0.04826000<br>C 2.91951300 1.71672300 -0.24817800<br>C -0.31670800 -1.16635500 -0.43985100<br>C -0.84377100 1.65936000 0.20014000	Zero-point correction= 0.327800<br>(Hartree/Particle)<br>Thermal correction to Energy= 0.348589<br>Thermal correction to Enthalpy= 0.349533<br>Thermal correction to Gibbs Free Energy= 0.276847<br>Sum of electronic and zero-point Energies= -<br>1109.059528<br>Sum of electronic and thermal Energies= -

C -1.94716200 0.63602100 0.27891400<br>C -1.69184000 -0.54795500 -0.65856000<br>C -2.77563200 -1.63547700 -0.50510500<br>H -2.44057900 -2.39607400 0.20629100<br>C -4.12042700 -1.10065000 -0.01829800<br>C -4.35090400 0.30924700 -0.54498900<br>C -3.31986600 1.29349200 0.02840500<br>H 3.70845600 2.45883800 -0.20491100<br>H -4.91769400 -1.76780600 -0.36279500<br>H -4.28085600 0.27298900 -1.63697300<br>H -3.68203800 1.71829500 0.96538700<br>H -3.19768100 2.13091400 -0.66176800<br>H -5.36298600 0.64828600 -0.30513900<br>H -2.91381000 -2.13694100 -1.46425600<br>H -0.32172100 -1.69411400 0.52803900<br>C 4.60808400 -0.10121100 -0.66396300<br>H 4.71774500 -0.57774100 -1.64129000<br>H 5.31993700 0.71956800 -0.58447700<br>O 2.42911600 -1.84101500 -0.72299600<br>H 2.73299600 -2.27669600 0.22138800<br>O -0.13438300 -2.09039200 -1.49723000<br>H 0.75330900 -2.45896600 -1.40978100<br>O -4.09075300 -1.11006300 1.41360000<br>H -4.92181200 -0.74461100 1.73509700<br>O 1.39262700 3.42788600 0.19533200<br>H 0.42039100 3.54792000 0.32674300<br>O -1.07091000 2.84706300 0.41875700<br>H -1.91303500 0.25536900 1.31038600<br>H -1.70413900 -0.16754700 -1.68809000<br>O 2.42704400 -1.34502400 2.05146400<br>H 3.16554200 -0.71907000 2.15721800<br>O 2.97204200 -2.46163800 1.51389600<br>H 4.85919100 -0.86150100 0.08220300	1109.038739<br>Sum of electronic and thermal Enthalpies= -1109.037795<br>Sum of electronic and thermal Free Energies= -1109.110481
**Name**	**1-C9-H-OOH**
Cartesian Coordinates	Frequency and Energy
C 3.42618400 -0.31483700 0.11717700<br>C 2.21229900 -0.93577800 -0.21374600<br>C 1.05581400 -0.19469800 -0.39213800<br>C 1.06077600 1.19030400 -0.12169800<br>C 2.27451300 1.82198700 0.19464200<br>C 3.44272700 1.05921300 0.29690700<br>C -0.17335100 -0.87961800 -0.89501400<br>C -0.21013800 1.92502400 -0.03655100<br>C -1.45321900 1.06748500 0.03816700<br>C -1.40513200 -0.01680900 -1.04811400<br>C -2.70538600 -0.86093300 -1.09314900<br>H -2.46398500 -1.92501000 -1.03851400<br>C -3.69984100 -0.52057200 0.00812800<br>C -3.99374800 0.98078800 0.07766400	Zero-point correction= 0.330145 (Hartree/Particle)<br>Thermal correction to Energy= 0.350519<br>Thermal correction to Enthalpy= 0.351463<br>Thermal correction to Gibbs Free Energy= 0.281324<br>Sum of electronic and zero-point Energies= -1109.025951<br>Sum of electronic and thermal Energies= -1109.005577<br>Sum of electronic and thermal Enthalpies= -1109.004632<br>Sum of electronic and thermal Free Energies= -1109.074771

C -2.74134700 1.87490900 -0.06776200<br>H 4.36313300 1.57204000 0.55034100<br>H -4.63118200 -1.06151700 -0.19036900<br>H -4.71224800 1.21606500 -0.71206900<br>H -2.73821100 2.67087800 0.67674400<br>H -2.74570300 2.37376700 -1.04173600<br>H -4.50756100 1.18800700 1.02199800<br>H -3.21323400 -0.70261600 -2.04723200<br>H -0.42324500 -1.72542000 -0.00137300<br>C 4.66340000 -1.15118300 0.26849400<br>H 5.51967800 -0.53310500 0.53607700<br>H 4.51648900 -1.91072400 1.03917200<br>H 4.88350300 -1.68234700 -0.65991100<br>O 2.19522800 -2.30433200 -0.39851200<br>H 1.50449200 -2.68268400 0.17267800<br>O 0.00250000 -1.64104900 -2.04354000<br>H 0.83369300 -2.12958400 -1.96847700<br>O -3.17171100 -1.00396000 1.25884400<br>H -3.81080300 -0.80532400 1.94968300<br>O 2.36306200 3.13853700 0.43070100<br>H 1.47681600 3.52956000 0.31445200<br>O -0.25681700 3.14382200 0.05688000<br>H -1.39880900 0.57375100 1.01729000<br>H -1.28955600 0.49267500 -2.01502700<br>O -0.66999200 -2.02178700 2.04447800<br>H -1.61685400 -1.79550200 1.99621900<br>O -0.47073600 -2.67805800 0.85139900	
**Name**	**2-O8-H-OOH**
Cartesian Coordinates	Frequency and Energy
C 3.04184600 0.49871300 -0.17733600<br>C 2.00576700 -0.46489200 -0.28931000<br>C 0.65137600 -0.07667900 -0.25085300<br>C 0.33573300 1.26652400 -0.02698500<br>C 1.37656600 2.22549600 0.08367200<br>C 2.72109600 1.82352700 -0.00704000<br>C -0.43057500 -1.13235800 -0.38910600<br>C -1.06503200 1.69549600 0.16424200<br>C -2.14480500 0.64236300 0.20281900<br>C -1.80388900 -0.54890200 -0.69735600<br>C -2.86723900 -1.66213400 -0.59622800<br>H -2.55834200 -2.40591700 0.14337000<br>C -4.24804000 -1.15286700 -0.18974900<br>C -4.48478100 0.24144900 -0.75809700<br>C -3.51612400 1.26071900 -0.13816200<br>H 3.48648400 2.58711200 0.06831600<br>H -5.00957800 -1.84957900 -0.55685000<br>H -4.34759800 0.19058000 -1.84317500<br>H -3.94260300 1.68618900 0.77056900<br>H -3.37337800 2.09683500 -0.82547300	Zero-point correction= 0.335087 (Hartree/Particle)<br>Thermal correction to Energy= 0.356486<br>Thermal correction to Enthalpy= 0.357430<br>Thermal correction to Gibbs Free Energy= 0.283891<br>Sum of electronic and zero-point Energies= -1184.238798<br>Sum of electronic and thermal Energies= -1184.217399<br>Sum of electronic and thermal Enthalpies= -1184.216455<br>Sum of electronic and thermal Free Energies= -1184.289993

H -5.51801600 0.55908400 -0.58943500<br>H -2.93140300 -2.17785400 -1.55542100<br>H -0.47977100 -1.65890900 0.57859800<br>C 4.47239400 0.01438800 -0.23467200<br>H 5.14983700 0.86665400 -0.17809000<br>H 4.66428100 -0.62926000 0.63550200<br>O 2.34562600 -1.75484200 -0.41470500<br>H 2.31663200 -2.22301300 0.57062900<br>O -0.16550300 -2.04446000 -1.43460800<br>H 0.71618000 -2.41036500 -1.30201500<br>O -4.28141600 -1.12943800 1.23900100<br>H -5.14993500 -0.83245100 1.52397100<br>O 1.13607600 3.51317200 0.27852100<br>H 0.15768300 3.63086100 0.32607400<br>O -1.33604800 2.88032400 0.32381800<br>H -2.16588200 0.27704000 1.24018700<br>H -1.75738900 -0.18776500 -1.73311800<br>O 4.76780000 -0.66474300 -1.43277800<br>H 4.20457600 -1.44576600 -1.46404600<br>O 1.57920500 -1.27805900 2.25009200<br>H 2.27724100 -0.71478400 2.62158900<br>O 2.20366300 -2.41492200 1.86488200	
**Name**	**2-O8-H-OOH (water)**
Cartesian Coordinates	Frequency and Energy
C 3.01647300 0.50518100 -0.21743900<br>C 1.98195400 -0.46199400 -0.31571200<br>C 0.62596700 -0.07675200 -0.26232300<br>C 0.30968100 1.26770100 -0.04382900<br>C 1.35357600 2.22419200 0.04910400<br>C 2.69645100 1.82996500 -0.05224000<br>C -0.45554300 -1.13539600 -0.35914300<br>C -1.09027600 1.68789400 0.16316800<br>C -2.16548900 0.63459700 0.21782100<br>C -1.83255500 -0.56643300 -0.67153900<br>C -2.89194700 -1.68032200 -0.54134200<br>H -2.57956800 -2.40025100 0.22153500<br>C -4.28508700 -1.17182400 -0.17603400<br>C -4.50547900 0.22215400 -0.74136000<br>C -3.54520600 1.24112100 -0.11159900<br>H 3.46749200 2.58798800 0.02592300<br>H -5.03567000 -1.86186600 -0.57176000<br>H -4.34881600 0.16835600 -1.82309600<br>H -3.97804900 1.65174800 0.80160000<br>H -3.41499600 2.07803400 -0.80034600<br>H -5.53958600 0.53778400 -0.58104700<br>H -2.95431300 -2.22300400 -1.48591400<br>H -0.49343300 -1.63769000 0.62015900<br>C 4.44616800 0.02927300 -0.24969900<br>H 5.11904300 0.88374300 -0.19253600	Zero-point correction= 0.332984 (Hartree/Particle)<br>Thermal correction to Energy= 0.354549<br>Thermal correction to Enthalpy= 0.355494<br>Thermal correction to Gibbs Free Energy= 0.281543<br>Sum of electronic and zero-point Energies= -1184.282306<br>Sum of electronic and thermal Energies= -1184.260741<br>Sum of electronic and thermal Enthalpies= -1184.259797<br>Sum of electronic and thermal Free Energies= -1184.333747

H 4.62573200 -0.61239800 0.62112000	
O 2.32292900 -1.75162800 -0.45174300	
H 2.36546700 -2.20511000 0.53754600	
O -0.18130500 -2.09014000 -1.37724900	
H 0.70466600 -2.44421200 -1.21778000	
O -4.38758200 -1.16006400 1.25759200	
H -5.24052600 -0.77452800 1.49089300	
O 1.11330000 3.52275200 0.24284600	
H 0.13763800 3.64229900 0.31279000	
O -1.36435300 2.87511500 0.33373200	
H -2.15497400 0.28722400 1.26184000	
H -1.80450900 -0.21229000 -1.70968800	
O 4.75763900 -0.66780600 -1.44961800	
H 4.21213000 -1.46472000 -1.46498300	
O 1.94729000 -1.16298500 2.27254300	
H 2.77810500 -0.68386800 2.44869000	
O 2.31483800 -2.39191600 1.84415200	
**Name**	**2-O8-H-OOH (pentyl ethanoate)**
Cartesian Coordinates	Frequency and Energy
C 3.03816100 0.50616500 -0.17332400	Zero-point correction= 0.333979
C 2.00282600 -0.45794900 -0.28855000	(Hartree/Particle)
C 0.64757000 -0.06975400 -0.25846600	Thermal correction to Energy= 0.355455
C 0.33144700 1.27201500 -0.03084200	Thermal correction to Enthalpy= 0.356399
C 1.37263700 2.22958800 0.08626400	Thermal correction to Gibbs Free Energy= 0.282794
C 2.71709400 1.83108500 -0.00260400	Sum of electronic and zero-point Energies= -
C -0.43379100 -1.12330000 -0.41266700	1184.270189
C -1.06963600 1.69755700 0.16243900	Sum of electronic and thermal Energies= -
C -2.14592500 0.64372800 0.20316300	1184.248712
C -1.81033400 -0.54096700 -0.70959400	Sum of electronic and thermal Enthalpies= -
C -2.87203400 -1.65682200 -0.60860400	1184.247768
H -2.55003500 -2.41199900 0.11438500	Sum of electronic and thermal Free Energies= -
C -4.25047400 -1.15943800 -0.18126100	1184.321374
C -4.49954900 0.23973700 -0.72735900	
C -3.52402900 1.25747300 -0.11655000	
H 3.48738800 2.58974400 0.07808700	
H -5.01248700 -1.85230100 -0.55360200	
H -4.38065100 0.19935800 -1.81493700	
H -3.94082000 1.67881900 0.79911200	
H -3.39459200 2.09281500 -0.80772800	
H -5.53054900 0.55002100 -0.53408300	
H -2.95491300 -2.15686100 -1.57488500	
H -0.48257000 -1.66882800 0.54347900	
C 4.47064600 0.03167400 -0.22541900	
H 5.14019000 0.89030300 -0.17585200	
H 4.67206500 -0.60320000 0.64777400	
O 2.34180300 -1.74790900 -0.42735500	
H 2.30746000 -2.22859400 0.54778300	
O -0.15674000 -2.02036200 -1.47408500	
H 0.72363800 -2.38695600 -1.33126600	

O -4.27914000 -1.15970100 1.25060900<br>H -5.13364700 -0.81874800 1.53552400<br>O 1.12421900 3.51679700 0.28450600<br>H 0.14242000 3.62084900 0.33215500<br>O -1.33844900 2.88413900 0.32925200<br>H -2.14665000 0.27399600 1.23915200<br>H -1.77573300 -0.16725400 -1.74087700<br>O 4.77085900 -0.65808500 -1.42343600<br>H 4.21482800 -1.44539500 -1.44260700<br>O 1.64777400 -1.29968200 2.27226600<br>H 2.39493200 -0.78035000 2.61998900<br>O 2.18618200 -2.46271800 1.84057300	
**Name**	**2-C9-H-OOH**
Cartesian Coordinates	Frequency and Energy
C 3.18517700 0.14760500 0.25586500<br>C 2.06201200 -0.61164300 -0.09755000<br>C 0.83887400 -0.00937100 -0.33939700<br>C 0.68512500 1.37339500 -0.10279500<br>C 1.80976200 2.14396500 0.23574000<br>C 3.05191400 1.51734200 0.39528500<br>C -0.29077100 -0.83734800 -0.85962700<br>C -0.66428100 1.96446500 -0.07323800<br>C -1.80414500 0.97357600 -0.00240400<br>C -1.60680600 -0.12190800 -1.05994300<br>C -2.80498100 -1.10607600 -1.11344600<br>H -2.44713500 -2.13624200 -1.04909700<br>C -3.84270300 -0.87090800 -0.02474200<br>C -4.31200700 0.58707600 0.02311700<br>C -3.17555200 1.61885000 -0.15617500<br>H 3.90448700 2.13331400 0.65742800<br>H -4.70167900 -1.52051500 -0.22287900<br>H -5.06439700 0.72384500 -0.75803400<br>H -3.27757000 2.44194300 0.55087300<br>H -3.22751400 2.06801900 -1.15287400<br>H -4.83136600 0.74968900 0.97303300<br>H -3.31697400 -1.00914500 -2.07354300<br>H -0.46501200 -1.69247000 0.04343400<br>C 4.50136300 -0.55566500 0.49426800<br>H 5.28171000 0.18426500 0.67369100<br>H 4.41065900 -1.17711400 1.39443700<br>O 2.20455400 -1.98343700 -0.23091100<br>H 1.52627200 -2.42133800 0.31502800<br>O -0.00240700 -1.59116900 -1.98935100<br>H 0.88088300 -1.97225000 -1.90000500<br>O -3.27276900 -1.27262100 1.23707000<br>H -3.94439200 -1.15933400 1.91656800<br>O 1.74586900 3.46648800 0.43733000<br>H 0.82912500 3.75797400 0.27680200<br>O -0.84733900 3.17148700 -0.01857900	Zero-point correction= 0.336127 (Hartree/Particle)<br>Thermal correction to Energy= 0.357352<br>Thermal correction to Enthalpy= 0.358296<br>Thermal correction to Gibbs Free Energy= 0.286731<br>Sum of electronic and zero-point Energies= -1184.235147<br>Sum of electronic and thermal Energies= -1184.213922<br>Sum of electronic and thermal Enthalpies= -1184.212978<br>Sum of electronic and thermal Free Energies= -1184.284543

H -1.71746000 0.51126500 0.98971800<br>H -1.52476100 0.37572900 -2.03634600<br>O -0.69290100 -1.98551100 2.09227500<br>H -1.65892500 -1.87261000 2.02533000<br>O -0.39829500 -2.62892000 0.91190100<br>O 4.92148100 -1.32957700 -0.60863700<br>H 4.28062800 -2.03907800 -0.72211000	
**Name**	**3-O8-H-OOH**
Cartesian Coordinates	Frequency and Energy
C 3.38650900 0.37992800 -0.50451000<br>C 2.33487300 -0.58134200 -0.47468700<br>C 0.99351700 -0.17736400 -0.33444700<br>C 0.69787300 1.18362800 -0.19245900<br>C 1.74577300 2.13702100 -0.26000200<br>C 3.07510400 1.71635700 -0.41073700<br>C -0.10342000 -1.23679600 -0.37681500<br>C -0.67627400 1.63246300 0.12286100<br>C -1.64544500 0.53468000 0.48334200<br>C -1.47063400 -0.59390100 -0.54511600<br>C -2.62130900 -1.58545300 -0.42428500<br>H -2.67438700 -1.94796500 0.60888600<br>C -3.96336900 -0.91717400 -0.76105700<br>C -3.92153300 0.60817600 -0.57154500<br>C -3.10860800 0.99382700 0.65995300<br>H 3.84690600 2.47627500 -0.43847000<br>H -4.23967100 -1.14349800 -1.79614400<br>H -3.47740900 1.07974200 -1.45366400<br>H -3.11421500 2.07645200 0.79130000<br>H -4.94102400 0.99496800 -0.48275500<br>H -2.44703700 -2.44927800 -1.06555700<br>H -0.08384700 -1.78888200 0.57908900<br>C 4.79562900 -0.11049300 -0.62316500<br>H 5.49400500 0.72104500 -0.70493300<br>H 5.05540500 -0.70702300 0.25621000<br>H 4.89944300 -0.76102900 -1.49407400<br>O 2.62263400 -1.87436500 -0.60796200<br>H 3.09509000 -2.24008600 0.30203200<br>O 0.06187800 -2.12893000 -1.45552500<br>H 0.94715600 -2.50785000 -1.38995100<br>O -4.92572100 -1.50777100 0.12208300<br>H -5.80861000 -1.24186000 -0.15048900<br>O 1.52194600 3.44267200 -0.16706500<br>H 0.55573400 3.58447600 -0.07330000<br>O -0.97923800 2.81731000 0.12460300<br>H -1.32123600 0.15484000 1.46293700<br>H -1.47480300 -0.16904400 -1.55856100<br>O -3.66232900 0.44075500 1.84506000<br>H -4.15158500 -0.36096400 1.62340800<br>O 3.22357300 -1.12254900 2.03636400	Zero-point correction= 0.334644 (Hartree/Particle)<br>Thermal correction to Energy= 0.355882<br>Thermal correction to Enthalpy= 0.356827<br>Thermal correction to Gibbs Free Energy= 0.284618<br>Sum of electronic and zero-point Energies= -1184.247852<br>Sum of electronic and thermal Energies= -1184.226614<br>Sum of electronic and thermal Enthalpies= -1184.225670<br>Sum of electronic and thermal Free Energies= -1184.297878

H 2.40672600 -1.17941500 2.55583400<br>O 3.42741200 -2.37865300 1.56546900	
**Name**	**3-O8-H-OOH (water)**
Cartesian Coordinates	Frequency and Energy
C 3.48957800 0.29653000 -0.42410800<br>C 2.40675500 -0.62543200 -0.46092000<br>C 1.07185800 -0.18070200 -0.37426700<br>C 0.81258000 1.18343700 -0.20310600<br>C 1.89426200 2.09701400 -0.20710500<br>C 3.21499400 1.64130200 -0.31873700<br>C -0.04856900 -1.20764600 -0.45859000<br>C -0.55426900 1.65508500 0.10000900<br>C -1.55112700 0.57437700 0.42809600<br>C -1.40224200 -0.53886200 -0.62497700<br>C -2.56925000 -1.51211900 -0.49595700<br>H -2.60614300 -1.87029700 0.53954000<br>C -3.90088300 -0.81984000 -0.81893300<br>C -3.83076500 0.69980200 -0.62257500<br>C -3.00158800 1.07094100 0.59861700<br>H 4.01545600 2.37193600 -0.29652600<br>H -4.19169400 -1.02953000 -1.85130500<br>H -3.37494100 1.15839800 -1.50452500<br>H -2.98906900 2.15252800 0.72897800<br>H -4.84400400 1.10027000 -0.53222700<br>H -2.42832500 -2.37985000 -1.14058500<br>H -0.04556300 -1.79079100 0.47570100<br>C 4.88852000 -0.22783200 -0.48853200<br>H 5.60903100 0.58671200 -0.42998700<br>H 5.06590600 -0.92383300 0.33615100<br>H 5.04787600 -0.78235400 -1.41644800<br>O 2.66671200 -1.93235200 -0.58514400<br>H 2.69226400 -2.37553900 0.40370000<br>O 0.13322300 -2.07745600 -1.56825600<br>H 0.99539200 -2.50281900 -1.46151100<br>O -4.87829900 -1.39888000 0.06341000<br>H -5.74143900 -1.02900000 -0.15892200<br>O 1.70985300 3.41665800 -0.08117800<br>H 0.74658300 3.58874500 -0.00816000<br>O -0.82501700 2.85165300 0.12764800<br>H -1.22511900 0.16266000 1.39414600<br>H -1.41318500 -0.09026800 -1.62674200<br>O -3.58330600 0.54193000 1.79646000<br>H -4.04393600 -0.28039100 1.57763700<br>O 2.38967800 -1.28663700 2.14511000<br>H 1.42338000 -1.20213400 2.24478900<br>O 2.61070700 -2.55251300 1.72104900	Zero-point correction= 0.332795 (Hartree/Particle)<br>Thermal correction to Energy= 0.354236<br>Thermal correction to Enthalpy= 0.355180<br>Thermal correction to Gibbs Free Energy= 0.282466<br>Sum of electronic and zero-point Energies= -1184.291098<br>Sum of electronic and thermal Energies= -1184.269658<br>Sum of electronic and thermal Enthalpies= -1184.268714<br>Sum of electronic and thermal Free Energies= -1184.341427
**Name**	**3-O8-H-OOH (pentyl ethanoate)**
Cartesian Coordinates	Frequency and Energy
C 3.40410500 0.37666800 -0.49966500	Zero-point correction= 0.333717

C 2.34918800 -0.58073500 -0.47728200<br>C 1.00673900 -0.17437100 -0.34198100<br>C 0.71562800 1.18607800 -0.18893700<br>C 1.76839700 2.13525600 -0.24314500<br>C 3.09672300 1.71439300 -0.39571000<br>C -0.09370600 -1.22907000 -0.39039100<br>C -0.65735500 1.63586500 0.12707700<br>C -1.63561400 0.54169600 0.46923300<br>C -1.46035600 -0.58628900 -0.56138900<br>C -2.60975800 -1.57935600 -0.42936700<br>H -2.65192600 -1.93365400 0.60728400<br>C -3.95352300 -0.91611200 -0.76738600<br>C -3.90734000 0.61001200 -0.59975100<br>C -3.09868400 1.00912100 0.62908500<br>H 3.87536500 2.46817100 -0.41596700<br>H -4.23894800 -1.15479700 -1.79594600<br>H -3.45133400 1.06174300 -1.48581900<br>H -3.10468100 2.09290900 0.74790600<br>H -4.92588100 1.00230200 -0.52669600<br>H -2.44122500 -2.44855000 -1.06516700<br>H -0.07626500 -1.78343500 0.56267300<br>C 4.81276500 -0.10985200 -0.62683700<br>H 5.51185000 0.72532900 -0.64684700<br>H 5.06495600 -0.76146700 0.21484300<br>H 4.93267900 -0.70180300 -1.53747600<br>O 2.63631300 -1.87549100 -0.61715600<br>H 3.02789700 -2.26233100 0.31724300<br>O 0.08200400 -2.12126500 -1.47376000<br>H 0.96674300 -2.49905800 -1.39304100<br>O -4.91558000 -1.49256200 0.13120500<br>H -5.79750500 -1.21905200 -0.14520600<br>O 1.54249400 3.44124600 -0.13687000<br>H 0.57335700 3.57271700 -0.04014700<br>O -0.94852000 2.82570400 0.14914600<br>H -1.31494800 0.15575500 1.44759300<br>H -1.47323800 -0.15758200 -1.57218800<br>O -3.66789600 0.47239200 1.82083300<br>H -4.15552500 -0.32990000 1.59374300<br>O 3.11368000 -1.17345800 2.07427000<br>H 2.24426800 -1.17915100 2.51127600<br>O 3.28389100 -2.43256200 1.60240900	(Hartree/Particle)<br>Thermal correction to Energy= 0.355121<br>Thermal correction to Enthalpy= 0.356065<br>Thermal correction to Gibbs Free Energy= 0.283404<br>Sum of electronic and zero-point Energies= -<br>1184.279376<br>Sum of electronic and thermal Energies= -<br>1184.257972<br>Sum of electronic and thermal Enthalpies= -<br>1184.257028<br>Sum of electronic and thermal Free Energies= -<br>1184.329689
**Name**	**3-C9-H-OOH**
Cartesian Coordinates	Frequency and Energy
C 3.75262500 -0.03849300 0.02658000<br>C 2.64177700 -0.86617000 -0.19519500<br>C 1.35678200 -0.35069300 -0.30961200<br>C 1.17073800 1.04566300 -0.13653600<br>C 2.28238200 1.88086000 0.09264200<br>C 3.56052600 1.32398200 0.16540700	Zero-point correction= 0.334921<br>(Hartree/Particle)<br>Thermal correction to Energy= 0.356248<br>Thermal correction to Enthalpy= 0.357192<br>Thermal correction to Gibbs Free Energy= 0.286100<br>Sum of electronic and zero-point Energies= -

C 0.18791700 -1.25083800 -0.55035600<br>C -0.17823300 1.61740900 -0.10468200<br>C -1.35517300 0.67963900 -0.14168600<br>C -1.09954600 -0.57563700 -0.97802700<br>C -2.28750900 -1.56843000 -0.90464400<br>H -2.04850500 -2.39286200 -0.22917100<br>C -3.58726200 -0.91133600 -0.44277400<br>C -3.71669500 0.47038400 -1.06794100<br>C -2.63801400 1.42422100 -0.54778800<br>H 4.39760200 1.98848000 0.34221400<br>H -4.43207800 -1.53974200 -0.73775200<br>H -3.63236900 0.36070000 -2.15257300<br>H -2.39603900 2.16069500 -1.32087500<br>H -4.69111500 0.91878500 -0.85880100<br>H -2.44566000 -2.01127100 -1.88973800<br>H -0.07294800 -1.73494100 0.61174800<br>C 5.12624200 -0.64530500 0.11769400<br>H 5.21354800 -1.30853100 0.98576500<br>H 5.36052600 -1.22685900 -0.77828600<br>H 5.88266200 0.13002800 0.22679000<br>O 2.80613800 -2.22863900 -0.32233300<br>H 3.61446600 -2.51685800 0.10960500<br>O 0.39130900 -2.33903000 -1.38078400<br>H 1.22655300 -2.76460800 -1.15050400<br>O -3.58621300 -0.83162500 0.99221800<br>H -3.86979300 0.04937500 1.26765000<br>O 2.18015400 3.20473200 0.25499000<br>H 1.23401600 3.44532700 0.20011200<br>O -0.37169900 2.82588500 0.03054100<br>H -1.47327200 0.36939100 0.90398400<br>H -0.95557100 -0.26902500 -2.02458400<br>O -1.19458100 -1.42950600 2.38353200<br>H -2.10069800 -1.46509300 2.01667000<br>O -0.48339700 -2.34467500 1.64639800<br>O -3.18763300 2.09729300 0.58081000<br>H -2.54327600 2.75999300 0.85628300	1184.247409<br>Sum of electronic and thermal Energies= -<br>1184.226082<br>Sum of electronic and thermal Enthalpies= -<br>1184.225138<br>Sum of electronic and thermal Free Energies= -<br>1184.296230
**Name**	**3- C9-H-OOH (water)**
Cartesian Coordinates	Frequency and Energy
C 3.73477600 -0.07739200 0.06686900<br>C 2.60921200 -0.88245800 -0.17916300<br>C 1.33785500 -0.33697700 -0.31247000<br>C 1.16702500 1.05942100 -0.12978200<br>C 2.29299900 1.86628200 0.10628000<br>C 3.55931300 1.28958700 0.19615500<br>C 0.16111200 -1.20872400 -0.61286000<br>C -0.17619800 1.64734100 -0.11829300<br>C -1.35998400 0.71566800 -0.12171000<br>C -1.11421500 -0.49931400 -1.02297100<br>C -2.31049900 -1.48380800 -1.03542900	Zero-point correction= 0.332236<br>(Hartree/Particle)<br>Thermal correction to Energy= 0.353893<br>Thermal correction to Enthalpy= 0.354837<br>Thermal correction to Gibbs Free Energy= 0.282821<br>Sum of electronic and zero-point Energies= -<br>1184.289732<br>Sum of electronic and thermal Energies= -<br>1184.268075<br>Sum of electronic and thermal Enthalpies= -<br>1184.267131

H -2.04978200 -2.40601800 -0.51220000<br>C -3.57699900 -0.91947700 -0.40802100<br>C -3.80258300 0.52079900 -0.83953000<br>C -2.65084900 1.46541500 -0.47550800<br>H 4.41130300 1.93240400 0.38583100<br>H -4.42856600 -1.52793900 -0.72355200<br>H -3.94319100 0.51385300 -1.92344800<br>H -2.44473000 2.12071900 -1.32751900<br>H -4.72337100 0.91001900 -0.39737200<br>H -2.54147300 -1.75539600 -2.06647700<br>H -0.11180800 -1.75042600 0.48416900<br>C 5.09261900 -0.70380600 0.18910500<br>H 5.10687900 -1.44364500 0.99504300<br>H 5.37152400 -1.21786400 -0.73686000<br>H 5.84573100 0.05370900 0.39913200<br>O 2.72817500 -2.24950900 -0.26964300<br>H 3.65130900 -2.51723100 -0.36674700<br>O 0.38848900 -2.25887900 -1.50520100<br>H 1.24592200 -2.66205400 -1.29848400<br>O -3.45347500 -1.03211100 1.02253100<br>H -4.19027800 -0.56993200 1.44094500<br>O 2.21248400 3.20891000 0.26772800<br>H 1.27328700 3.46726700 0.17329500<br>O -0.34350800 2.86694900 -0.03950900<br>H -1.44133800 0.37165200 0.91615500<br>H -0.94878900 -0.12819900 -2.04349900<br>O -1.13456700 -1.56051800 2.37342700<br>H -2.02613700 -1.42756400 1.97548200<br>O -0.51487300 -2.44824200 1.52607800<br>O -3.06661200 2.27321100 0.62878500<br>H -2.36690600 2.92281500 0.77650000	Sum of electronic and thermal Free Energies= -1184.339147
**Name**	**3- C9-H-OOH (pentyl ethanoate)**
Cartesian Coordinates	Frequency and Energy
C 3.76020200 -0.04936300 0.03126400<br>C 2.64334300 -0.87355000 -0.18769400<br>C 1.35967500 -0.35197900 -0.29785200<br>C 1.17759100 1.04524300 -0.12273500<br>C 2.29388300 1.87454600 0.09720800<br>C 3.57075100 1.31463200 0.16264000<br>C 0.18517400 -1.24452200 -0.54742600<br>C -0.16849300 1.62248800 -0.09138400<br>C -1.35006700 0.69105700 -0.13414200<br>C -1.09418400 -0.55656100 -0.98411500<br>C -2.28934900 -1.54244000 -0.94472200<br>H -2.06145100 -2.39141400 -0.29624100<br>C -3.58663300 -0.89461800 -0.46958400<br>C -3.71577200 0.50057400 -1.05907100<br>C -2.63096500 1.44318400 -0.53385400<br>H 4.41572300 1.97100000 0.33557400	Zero-point correction= 0.333976 (Hartree/Particle)<br>Thermal correction to Energy= 0.355372<br>Thermal correction to Enthalpy= 0.356316<br>Thermal correction to Gibbs Free Energy= 0.285130<br>Sum of electronic and zero-point Energies= -1184.278297<br>Sum of electronic and thermal Energies= -1184.256901<br>Sum of electronic and thermal Enthalpies= -1184.255956<br>Sum of electronic and thermal Free Energies= -1184.327142

H -4.43192000 -1.51416600 -0.77925900<br>H -3.64078500 0.41262300 -2.14605300<br>H -2.38747200 2.18022100 -1.30510200<br>H -4.69013200 0.94217500 -0.83439400<br>H -2.45336200 -1.94881900 -1.94424800<br>H -0.09277200 -1.73447600 0.59847000<br>C 5.13070500 -0.65786800 0.12948500<br>H 5.19554000 -1.35334400 0.97308000<br>H 5.39204600 -1.20317200 -0.78354500<br>H 5.88225000 0.11580000 0.27970200<br>O 2.78941700 -2.23464500 -0.28447900<br>H 3.71499900 -2.49335900 -0.20809500<br>O 0.39568000 -2.33078900 -1.37894500<br>H 1.24950900 -2.73013600 -1.16511500<br>O -3.58354600 -0.85084600 0.97231500<br>H -3.90085900 0.01234700 1.26727300<br>O 2.19122300 3.20167300 0.25782000<br>H 1.24103800 3.43323000 0.20800700<br>O -0.35288300 2.83359100 0.04502900<br>H -1.46833600 0.37527000 0.90971400<br>H -0.93445300 -0.23195500 -2.02183300<br>O -1.25085100 -1.48878200 2.38282000<br>H -2.13458200 -1.45876000 1.95912300<br>O -0.52933700 -2.38211300 1.62580800<br>O -3.17146000 2.11746100 0.60232900<br>H -2.51609500 2.76759900 0.88325600		
**Name**	**4-O8-H-OOH**	
Cartesian Coordinates	Frequency and Energy	
C 3.33235200 0.52690800 -0.47483100<br>C 2.32999000 -0.47990600 -0.56607900<br>C 0.97122300 -0.16615200 -0.35890600<br>C 0.61673400 1.13992800 -0.00080800<br>C 1.62365800 2.13786000 0.09460700<br>C 2.96641200 1.81473500 -0.15654800<br>C -0.06894300 -1.26791000 -0.45084300<br>C -0.77189700 1.48006800 0.34532700<br>C -1.81629100 0.39497000 0.34252300<br>C -1.48800900 -0.74246600 -0.62160500<br>C -2.48892500 -1.90605100 -0.46509900<br>H -2.08153300 -2.66110100 0.21293500<br>C -3.84367300 -1.46877800 0.08700300<br>C -4.20131000 -0.08031300 -0.42095000<br>C -3.22053100 0.99226100 0.06651700<br>H 3.70098600 2.60726500 -0.07522800<br>H -4.60639500 -2.18926800 -0.22766500<br>H -4.16360500 -0.08536400 -1.51492900<br>H -3.58240100 1.44048400 0.99681900<br>H -5.22026100 0.19986000 -0.14016300<br>H -2.62605000 -2.38953800 -1.43329400	Zero-point correction= 0.334773 (Hartree/Particle)<br>Thermal correction to Energy= 0.355925<br>Thermal correction to Enthalpy= 0.356869<br>Thermal correction to Gibbs Free Energy= 0.284751<br>Sum of electronic and zero-point Energies= -1184.248316<br>Sum of electronic and thermal Energies= -1184.227164<br>Sum of electronic and thermal Enthalpies= -1184.226220<br>Sum of electronic and thermal Free Energies= -1184.298339	

H -0.00849700 -1.82496200 0.50014000<br>C 4.75943800 0.12969900 -0.70304300<br>H 5.42562600 0.98604000 -0.60745700<br>H 5.06028300 -0.64170400 0.01232700<br>O 2.70773300 -1.73187700 -0.81790600<br>H 2.98154300 -2.20642600 0.13054600<br>O 0.14221300 -2.13489000 -1.54365500<br>H 1.05338400 -2.44875700 -1.50638800<br>O -3.74841300 -1.48203700 1.51533300<br>H -4.60803300 -1.26096600 1.88365600<br>O 1.35343900 3.39242000 0.42773700<br>H 0.38596300 3.45672900 0.59890400<br>O -1.07252100 2.62939200 0.67178900<br>H -1.82242000 -0.00780500 1.36609900<br>H -1.55032000 -0.34162200 -1.64088300<br>O 2.42888100 -1.40991800 1.95135100<br>H 3.06831900 -0.71698200 2.18165700<br>O 3.16480800 -2.41003600 1.40872200<br>H 4.87437100 -0.31073500 -1.69566300<br>O -3.19302200 1.97379800 -0.95598700<br>H -2.74728000 2.75090700 -0.60407500	
**Name**	**4-O8-H-OOH (water)**
Cartesian Coordinates	Frequency and Energy
C 3.31068100 0.54906900 -0.49643900<br>C 2.31202100 -0.46189800 -0.57457100<br>C 0.95170200 -0.15960100 -0.35828200<br>C 0.59220500 1.14597600 -0.00302700<br>C 1.59722500 2.14814100 0.06926200<br>C 2.93668700 1.83821600 -0.19263000<br>C -0.08343500 -1.26769600 -0.42169600<br>C -0.79216100 1.47154000 0.36823400<br>C -1.83534800 0.38653200 0.35300800<br>C -1.50605700 -0.75789100 -0.60174800<br>C -2.50247600 -1.92197500 -0.42394800<br>H -2.09702000 -2.65249400 0.28271300<br>C -3.87622100 -1.48264800 0.08081800<br>C -4.21341000 -0.09197400 -0.43025700<br>C -3.23982900 0.97719600 0.07333700<br>H 3.67338900 2.62968700 -0.11868300<br>H -4.63061400 -2.19164700 -0.27017600<br>H -4.15452100 -0.11608300 -1.52330600<br>H -3.61293600 1.42435900 0.99725200<br>H -5.23549100 0.18470200 -0.16174900<br>H -2.62962000 -2.43458500 -1.37857200<br>H -0.01931400 -1.80982400 0.53476600<br>C 4.74013100 0.16275700 -0.70563200<br>H 5.39552000 1.02319800 -0.58008900<br>H 5.02556900 -0.61489900 0.00971700<br>O 2.69218100 -1.71635700 -0.85080300	Zero-point correction= 0.332119 (Hartree/Particle)<br>Thermal correction to Energy= 0.353658<br>Thermal correction to Enthalpy= 0.354602<br>Thermal correction to Gibbs Free Energy= 0.281383<br>Sum of electronic and zero-point Energies= -1184.290489<br>Sum of electronic and thermal Energies= -1184.268950<br>Sum of electronic and thermal Enthalpies= -1184.268006<br>Sum of electronic and thermal Free Energies= -1184.341225

H 2.96843500 -2.20766700 0.07476300<br>O 0.14593000 -2.16751000 -1.49800000<br>H 1.06706800 -2.45875300 -1.44155900<br>O -3.85717900 -1.51063100 1.51702700<br>H -4.72166800 -1.21932800 1.83025000<br>O 1.31849000 3.41331500 0.39637400<br>H 0.35633000 3.47304500 0.59320200<br>O -1.09579900 2.61127100 0.73027400<br>H -1.83506300 -0.00709600 1.37948300<br>H -1.57637500 -0.36771100 -1.62467000<br>O 2.82257900 -1.31979900 1.94823200<br>H 3.64431100 -0.79829900 2.00679000<br>O 3.16953200 -2.48647100 1.35793900<br>H 4.88182000 -0.25595800 -1.70492400<br>O -3.20583000 1.97958900 -0.94438800<br>H -2.76934500 2.75765300 -0.57702300	
**Name**	**4-O8-H-OOH (pentyl ethanoate)**
Cartesian Coordinates	Frequency and Energy
C 3.31801300 0.54710700 -0.48934300<br>C 2.32252300 -0.46901300 -0.56150600<br>C 0.96099400 -0.16506500 -0.35305100<br>C 0.60013600 1.13981400 0.00220700<br>C 1.60217500 2.14420000 0.08924100<br>C 2.94361500 1.83395400 -0.17515000<br>C -0.07597200 -1.27101500 -0.44194900<br>C -0.78788000 1.47189200 0.35407000<br>C -1.82993200 0.38633600 0.34518500<br>C -1.49860100 -0.75469500 -0.61522800<br>C -2.49605400 -1.91971200 -0.44755500<br>H -2.08836800 -2.66320100 0.24353500<br>C -3.86004000 -1.48372500 0.08436300<br>C -4.21034300 -0.09564000 -0.42820800<br>C -3.23795500 0.97682800 0.07250200<br>H 3.67711500 2.62909500 -0.10766100<br>H -4.61789300 -2.20128700 -0.24538200<br>H -4.15826700 -0.10998000 -1.52168300<br>H -3.60651300 1.41278700 1.00522100<br>H -5.23284800 0.18081700 -0.15763600<br>H -2.62971900 -2.41699500 -1.40931700<br>H -0.01775100 -1.82824800 0.50761000<br>C 4.74696700 0.17377600 -0.73692400<br>H 5.39577800 1.04472000 -0.65231500<br>H 5.07994100 -0.58643900 -0.02341100<br>O 2.70411900 -1.72043400 -0.82523500<br>H 3.01337000 -2.19184600 0.10336500<br>O 0.14861000 -2.14378600 -1.53313000<br>H 1.06644800 -2.43856400 -1.48722700<br>O -3.79522400 -1.49974100 1.51625000<br>H -4.65836100 -1.24549200 1.85988100	Zero-point correction= 0.333146 (Hartree/Particle)<br>Thermal correction to Energy= 0.354701<br>Thermal correction to Enthalpy= 0.355646<br>Thermal correction to Gibbs Free Energy= 0.282169<br>Sum of electronic and zero-point Energies= -1184.279030<br>Sum of electronic and thermal Energies= -1184.257474<br>Sum of electronic and thermal Enthalpies= -1184.256530<br>Sum of electronic and thermal Free Energies= -1184.330006

O 1.31828200 3.39636100 0.42500000<br>H 0.34976600 3.44144700 0.60545000<br>O -1.08998100 2.61872700 0.69393800<br>H -1.83086900 -0.01311100 1.36995800<br>H -1.56563100 -0.35953200 -1.63623600<br>O 2.62353900 -1.37958600 1.96943200<br>H 3.32878300 -0.72712800 2.12840100<br>O 3.23429700 -2.43643200 1.38349200<br>H 4.85927400 -0.26260600 -1.73229700<br>O -3.21578100 1.97690100 -0.93860500<br>H -2.77300800 2.74923000 -0.57133000	
**Name**	**4-C9-H-OOH**
Cartesian Coordinates	Frequency and Energy
C -3.58399300 -0.07051900 -0.06703400<br>C -2.43234700 -0.79930200 0.27215000<br>C -1.20058500 -0.17756200 0.38164700<br>C -1.06927000 1.18346600 0.02791300<br>C -2.21865400 1.92453600 -0.29337900<br>C -3.46279700 1.28560100 -0.32435300<br>C -0.03477700 -0.95652800 0.90040000<br>C 0.26622600 1.76316500 -0.12762500<br>C 1.40615100 0.77122600 -0.19274300<br>C 1.28785900 -0.22600500 0.96945100<br>C 2.48797400 -1.20488000 1.02825700<br>H 2.13274100 -2.23787800 1.02413200<br>C 3.48782600 -1.02057300 -0.10406800<br>C 3.92187500 0.43665700 -0.25275300<br>C 2.77087200 1.45991900 -0.21320500<br>H -4.33193600 1.87802700 -0.58487200<br>H 4.36788200 -1.63518200 0.11053200<br>H 4.59212600 0.68295100 0.57462100<br>H 2.81551300 2.11004400 -1.09190800<br>H 4.50387000 0.54756600 -1.17244400<br>H 3.03041000 -1.05678600 1.96405400<br>H 0.09669600 -1.87259800 0.05347100<br>C -4.90506800 -0.77881100 -0.14248900<br>H -5.70247600 -0.08656000 -0.40966600<br>H -4.86331300 -1.57934500 -0.88406000<br>H -5.14408000 -1.24759800 0.81423400<br>O -2.55544600 -2.14846500 0.53472200<br>H -1.92298900 -2.62899100 -0.02725900<br>O -0.25612100 -1.62325200 2.09765100<br>H -1.13388800 -2.02773400 2.07397100<br>O 2.89477300 -1.51464300 -1.32277300<br>H 3.55191800 -1.45501200 -2.02262300<br>O -2.17577500 3.22806100 -0.60288600<br>H -1.25445700 3.53421800 -0.52150900<br>O 0.45039700 2.96902100 -0.26970000<br>H 1.26972400 0.22679100 -1.13594500	Zero-point correction= 0.335132 (Hartree/Particle)<br>Thermal correction to Energy= 0.356508<br>Thermal correction to Enthalpy= 0.357452<br>Thermal correction to Gibbs Free Energy= 0.285759<br>Sum of electronic and zero-point Energies= -1184.246314<br>Sum of electronic and thermal Energies= -1184.224938<br>Sum of electronic and thermal Enthalpies= -1184.223994<br>Sum of electronic and thermal Free Energies= -1184.295687

H	1.26905600	0.35408600	1.89960700	
O	0.27372800	-2.27829300	-1.98199700	
H	1.24243500	-2.16832700	-1.96173400	
O	0.02254000	-2.85925800	-0.75989000	
O	2.94849500	2.24395200	0.95801500	
H	2.33228700	2.98301900	0.89924900	
**Name**				**4- C9-H-OOH (water)**
Cartesian Coordinates				Frequency and Energy
C	-3.68368900	-0.12753600	0.00156400	Zero-point correction= 0.332313 (Hartree/Particle)
C	-2.53284900	-0.87256800	0.31003100	
C	-1.27064700	-0.29136800	0.32516900	Thermal correction to Energy= 0.353940
C	-1.13554100	1.07026400	-0.04627500	Thermal correction to Enthalpy= 0.354884
C	-2.28636700	1.81980000	-0.34277900	Thermal correction to Gibbs Free Energy= 0.282740
C	-3.54175400	1.21375100	-0.31170700	Sum of electronic and zero-point Energies= -1184.288395
C	-0.07224100	-1.09720200	0.71362900	
C	0.19417800	1.67464700	-0.18430000	Sum of electronic and thermal Energies= -1184.266769
C	1.38879300	0.75738800	-0.13432900	
C	1.21008500	-0.32193100	0.93863700	Sum of electronic and thermal Enthalpies= -1184.265824
C	2.43605200	-1.26581000	1.03321300	
H	2.14316900	-2.29319100	0.80667300	Sum of electronic and thermal Free Energies= -1184.337969
C	3.59227700	-0.88259900	0.12121400	
C	3.91080100	0.60456000	0.19938500	
C	2.69858400	1.53602400	0.02938100	
H	-4.41353600	1.81199500	-0.55114100	
H	4.47746400	-1.44499500	0.42933800	
H	4.33644800	0.79100200	1.18907000	
H	2.83158800	2.16528000	-0.85301700	
H	4.68454300	0.84763000	-0.53234300	
H	2.81630600	-1.26183600	2.05612300	
H	0.13919000	-1.82650700	-0.27825900	
C	-5.03148500	-0.78722800	0.01670900	
H	-5.81009700	-0.07335800	-0.24686700	
H	-5.06614000	-1.61695500	-0.69565600	
H	-5.25829200	-1.19194600	1.00861800	
O	-2.61295500	-2.21493900	0.59298700	
H	-3.53014200	-2.49833000	0.69872300	
O	-0.25335700	-1.97504800	1.78679700	
H	-1.10151500	-2.43085400	1.67383700	
O	3.26603600	-1.28538000	-1.22385300	
H	3.97591200	-1.00229000	-1.81317600	
O	-2.23973800	3.13177700	-0.67881800	
H	-1.30426100	3.41643400	-0.64482900	
O	0.33335600	2.88114500	-0.39741400	
H	1.40402000	0.27817500	-1.12108700	
H	1.08568600	0.19085600	1.90095200	
O	0.86176800	-1.91316600	-2.28794500	
H	1.78848100	-1.68603200	-2.04352900	
O	0.43999400	-2.68123500	-1.22908300	
O	2.64771700	2.37820900	1.18628800	

H 2.00143000 3.07286800 1.00764600	
**Name**	**4- C9-H-OOH (pentyl ethanoate)**
Cartesian Coordinates	Frequency and Energy
C -3.59037600 -0.07998100 -0.07022400<br>C -2.43447300 -0.80295700 0.26826400<br>C -1.20320500 -0.17685100 0.37337100<br>C -1.07737600 1.18415700 0.01741400<br>C -2.23094300 1.91811900 -0.30321300<br>C -3.47300600 1.27688300 -0.33122100<br>C -0.03126200 -0.94822900 0.89339500<br>C 0.25667500 1.77309100 -0.13560500<br>C 1.40182300 0.79092500 -0.19620600<br>C 1.28345600 -0.20292400 0.97017900<br>C 2.50006000 -1.15873100 1.05250900<br>H 2.16429200 -2.19732200 1.09659800<br>C 3.48462200 -1.00212200 -0.09592200<br>C 3.91174600 0.45181900 -0.29089400<br>C 2.76668400 1.47783000 -0.22204400<br>H -4.34858400 1.86153100 -0.59024100<br>H 4.37013400 -1.60504200 0.12397600<br>H 4.62325000 0.70675100 0.49844600<br>H 2.80639900 2.13742300 -1.09314900<br>H 4.44772600 0.54117800 -1.23975900<br>H 3.05219600 -0.96282700 1.97391200<br>H 0.11186400 -1.86865800 0.05181000<br>C -4.91146700 -0.78623700 -0.14196500<br>H -5.70649900 -0.09218500 -0.41310500<br>H -4.87441200 -1.58991000 -0.88137200<br>H -5.15570500 -1.24694400 0.81812500<br>O -2.55164900 -2.15068400 0.53670200<br>H -1.90945900 -2.63178200 -0.01285300<br>O -0.25466700 -1.61435500 2.09073700<br>H -1.13263000 -2.01953100 2.06954500<br>O 2.88019600 -1.53222500 -1.29540800<br>H 3.52847500 -1.47458800 -2.00714000<br>O -2.18917200 3.22374100 -0.61391600<br>H -1.26553600 3.52557200 -0.52995500<br>O 0.42730500 2.98198700 -0.27558700<br>H 1.27030200 0.24663700 -1.14010700<br>H 1.24064400 0.38258600 1.89629700<br>O 0.33194900 -2.36950100 -1.96952900<br>H 1.29266100 -2.19508900 -1.90957500<br>O 0.05606800 -2.89241200 -0.72576600<br>O 2.95442000 2.25402700 0.95866400<br>H 2.33153500 2.98882700 0.91434700	Zero-point correction= 0.333848 (Hartree/Particle)<br>Thermal correction to Energy= 0.355449<br>Thermal correction to Enthalpy= 0.356393<br>Thermal correction to Gibbs Free Energy= 0.283834<br>Sum of electronic and zero-point Energies= -1184.274698<br>Sum of electronic and thermal Energies= -1184.253097<br>Sum of electronic and thermal Enthalpies= -1184.252153<br>Sum of electronic and thermal Free Energies= -1184.324712
**Name**	**5-O5-H-OOH**
Cartesian Coordinates	Frequency and Energy
C -3.50091100 1.14804200 -0.31048700<br>C -2.24461300 1.79453300 -0.23213900	Zero-point correction= 0.362439 (Hartree/Particle)

C -1.04695700 1.04838300 -0.10861500<br>C -1.07438800 -0.35361500 -0.10902300<br>C -2.33605600 -1.00173200 -0.14957800<br>C -3.51680300 -0.22133000 -0.24613200<br>C 0.22214100 1.77483600 0.09544500<br>C 0.21473200 -1.16044700 -0.03387800<br>C 1.46296700 -0.33320200 -0.37136000<br>C 1.44332800 0.96312200 0.45517900<br>C 2.71830800 1.79119900 0.31427800<br>H 2.67444100 2.66794100 0.96152700<br>C 3.95195800 0.95745900 0.63620500<br>C 3.99433800 -0.28182300 -0.24646800<br>C 2.74554800 -1.13583600 -0.08680400<br>H -4.45423200 -0.76578900 -0.28593300<br>H 3.91405800 0.65182000 1.68750200<br>H 4.07282500 0.01877400 -1.29980800<br>H 2.68987600 -1.48611300 0.95776300<br>H 4.87180400 -0.88711200 -0.01084900<br>H 2.79262600 2.15872400 -0.71741100<br>C -4.74109200 1.98284500 -0.43872800<br>H -5.62754200 1.35070900 -0.47586100<br>H -4.82913500 2.67278500 0.40325600<br>H -4.70097300 2.59284800 -1.34371300<br>O -2.25559500 3.12213200 -0.26656700<br>H -1.32393900 3.43549300 -0.20385100<br>O 5.13822000 1.72377600 0.52916800<br>H 5.25011400 1.98411300 -0.39011100<br>O -2.46830500 -2.30835800 -0.13242900<br>H -1.93859800 -2.72578600 0.72932800<br>O 0.15551500 -2.36059200 -0.78780400<br>H 1.45956700 -0.06948400 -1.43767500<br>H 1.31017900 0.67891900 1.51244200<br>C -0.12364000 -2.21977100 -2.17154000<br>H -1.01062000 -1.60361700 -2.34193400<br>H 0.73119600 -1.79615000 -2.70817600<br>H -0.31974300 -3.22348800 -2.54454600<br>H 0.31682300 -1.50810300 1.00172500<br>O 0.28048000 2.99448400 0.00794600<br>O 2.89310100 -2.23776000 -0.95733100<br>H 2.09930200 -2.78037800 -0.87372800<br>O -1.53731300 -2.87723800 1.96258300<br>O -1.53673300 -1.59937300 2.44766500<br>H -2.34615000 -1.53865300 2.97609400	Thermal correction to Energy= 0.385393<br>Thermal correction to Enthalpy= 0.386337<br>Thermal correction to Gibbs Free Energy= 0.310636<br>Sum of electronic and zero-point Energies= -1223.509021<br>Sum of electronic and thermal Energies= -1223.486068<br>Sum of electronic and thermal Enthalpies= -1223.485124<br>Sum of electronic and thermal Free Energies= -1223.560824
**Name**	**5-O5-H-OOH (water)**
Cartesian Coordinates	Frequency and Energy
C -3.49411800 1.07065700 -0.29036600<br>C -2.24279500 1.73135500 -0.28788500<br>C -1.03167200 1.00860700 -0.18654900<br>C -1.04868500 -0.39073900 -0.14500000	Zero-point correction= 0.360492 (Hartree/Particle)<br>Thermal correction to Energy= 0.383639<br>Thermal correction to Enthalpy= 0.384584

C -2.29999700 -1.04785800 -0.08921400<br>C -3.49467600 -0.29688900 -0.16742200<br>C 0.23802200 1.73742800 -0.00144100<br>C 0.23690100 -1.19711100 -0.14152300<br>C 1.49239600 -0.35643700 -0.40735400<br>C 1.43603900 0.93289900 0.42942800<br>C 2.71821200 1.75620700 0.35442500<br>H 2.64659700 2.61995900 1.01783100<br>C 3.92873100 0.91353200 0.72748100<br>C 4.01440000 -0.30995600 -0.17187100<br>C 2.75809800 -1.15905200 -0.07327700<br>H -4.43250500 -0.84135100 -0.13645500<br>H 3.84798800 0.59849000 1.77207000<br>H 4.13961400 0.02140900 -1.21036300<br>H 2.65699500 -1.53106400 0.95660400<br>H 4.88069900 -0.91801800 0.10042800<br>H 2.85331300 2.12706400 -0.66885700<br>C -4.75229300 1.87818500 -0.39431300<br>H -5.62344700 1.22451400 -0.37649200<br>H -4.82511900 2.58796300 0.43338400<br>H -4.76330700 2.45786800 -1.32047500<br>O -2.27044600 3.06462800 -0.36448900<br>H -1.34429900 3.39406900 -0.34474600<br>O 5.12753900 1.68517000 0.65947400<br>H 5.24651300 1.96539500 -0.25698100<br>O -2.38758800 -2.37160900 0.02254800<br>H -2.00642300 -2.66504100 0.98494100<br>O 0.18351600 -2.31386500 -1.03160400<br>H 1.53688200 -0.07796400 -1.46744200<br>H 1.24321000 0.64548200 1.47521600<br>C -0.24272000 -2.03620300 -2.36285000<br>H -1.32430900 -1.88276700 -2.40361000<br>H 0.26341500 -1.16188900 -2.77981600<br>H 0.01522800 -2.91266400 -2.95589100<br>H 0.33743100 -1.64935700 0.85268200<br>O 0.29988900 2.95506400 -0.14690600<br>O 2.92990200 -2.26319100 -0.96054100<br>H 2.06589900 -2.69009300 -1.06085000<br>O -1.65877700 -2.65034700 2.28586300<br>O -1.51483400 -1.31975400 2.51202100<br>H -2.38304100 -1.00851900 2.82428400	Thermal correction to Gibbs Free Energy= 0.308297<br>Sum of electronic and zero-point Energies= -1223.552130<br>Sum of electronic and thermal Energies= -1223.528983<br>Sum of electronic and thermal Enthalpies= -1223.528039<br>Sum of electronic and thermal Free Energies= -1223.604325
**Name**	**5-O5-H-OOH (pentyl ethanoate)**
Cartesian Coordinates	Frequency and Energy
C -3.49666700 1.14803400 -0.30296300<br>C -2.23846000 1.79246600 -0.23766500<br>C -1.03971000 1.04664200 -0.12190200<br>C -1.06915000 -0.35464800 -0.11974700<br>C -2.33191000 -0.99952300 -0.14264900<br>C -3.51330000 -0.22184000 -0.23230400	Zero-point correction= 0.361540 (Hartree/Particle)<br>Thermal correction to Energy= 0.384636<br>Thermal correction to Enthalpy= 0.385580<br>Thermal correction to Gibbs Free Energy= 0.309012<br>Sum of electronic and zero-point Energies= -

C 0.23166700 1.76961300 0.07984400<br>C 0.21771800 -1.16404900 -0.06390000<br>C 1.47062900 -0.33610300 -0.38275200<br>C 1.44651200 0.95723300 0.44926500<br>C 2.72607800 1.78030000 0.32188400<br>H 2.67874900 2.65308900 0.97483100<br>C 3.95067100 0.94058400 0.65933000<br>C 4.00075800 -0.29536500 -0.22706900<br>C 2.74614200 -1.14233300 -0.08094200<br>H -4.45542300 -0.75948700 -0.25855300<br>H 3.89928400 0.63321900 1.70901200<br>H 4.09478000 0.01327800 -1.27650700<br>H 2.67589400 -1.49166400 0.96178600<br>H 4.87288800 -0.90423600 0.02243700<br>H 2.81835600 2.14621600 -0.70868900<br>C -4.74075300 1.97635200 -0.42238000<br>H -5.62277800 1.33679300 -0.44824800<br>H -4.82903200 2.66728200 0.41961800<br>H -4.71703500 2.58142700 -1.33198500<br>O -2.24106100 3.12040700 -0.27590400<br>H -1.30319400 3.42109800 -0.21753000<br>O 5.14458500 1.70517600 0.56084400<br>H 5.24850700 1.97776200 -0.35790500<br>O -2.46094500 -2.31086100 -0.11667500<br>H -1.95572800 -2.71288700 0.74972300<br>O 0.15760400 -2.34630700 -0.85217700<br>H 1.47891400 -0.06508800 -1.44642700<br>H 1.30149000 0.67215700 1.50434400<br>C -0.15139000 -2.16989300 -2.22727800<br>H -1.10678100 -1.65639700 -2.36471500<br>H 0.63611000 -1.62114000 -2.75291200<br>H -0.22874000 -3.17018400 -2.65223700<br>H 0.32015500 -1.53744700 0.96244500<br>O 0.28825700 2.99118000 -0.01166100<br>O 2.89671300 -2.25378400 -0.94836400<br>H 2.06396400 -2.74320600 -0.92866400<br>O -1.57913800 -2.86671800 2.01808300<br>O -1.54826300 -1.58147000 2.47494300<br>H -2.38540100 -1.46639300 2.95482100	1223.540727<br>Sum of electronic and thermal Energies= -<br>1223.517631<br>Sum of electronic and thermal Enthalpies= -<br>1223.516687<br>Sum of electronic and thermal Free Energies= -<br>1223.593255
**Name**	**5-C10-H-OOH**
Cartesian Coordinates	Frequency and Energy
C 3.70551500 -0.68214300 -0.21557900<br>C 2.53062400 -1.46161200 -0.31902000<br>C 1.27303400 -0.85654300 -0.23831100<br>C 1.15796400 0.55498000 -0.10708200<br>C 2.32773300 1.30940000 0.03077300<br>C 3.57761200 0.67849300 -0.03419600<br>C 0.06642300 -1.69823200 -0.18760800<br>C -0.16939500 1.19658100 -0.05665100	Zero-point correction= 0.362551<br>(Hartree/Particle)<br>Thermal correction to Energy= 0.385549<br>Thermal correction to Enthalpy= 0.386493<br>Thermal correction to Gibbs Free Energy= 0.310864<br>Sum of electronic and zero-point Energies= -<br>1223.508348<br>Sum of electronic and thermal Energies= -

C -1.36552200 0.34522900 -0.44841500<br>C -1.22988000 -1.03284300 0.22353100<br>C -2.43079600 -1.93592700 -0.04238900<br>H -2.31381800 -2.88227000 0.48671300<br>C -3.72875900 -1.25505900 0.37283000<br>C -3.87623900 0.08480400 -0.33616600<br>C -2.70032700 1.00689600 -0.05537900<br>H 4.45878300 1.30409300 0.05888500<br>H -3.71418700 -1.08922800 1.45541500<br>H -3.93596000 -0.07745000 -1.42086200<br>H -2.66269700 1.22196800 1.02339500<br>H -4.79872600 0.57893100 -0.02558400<br>H -2.47194100 -2.17160600 -1.11421000<br>C 5.03736300 -1.36676200 -0.29542200<br>H 5.84970500 -0.64936400 -0.18388300<br>H 5.12325700 -2.12958300 0.48167600<br>H 5.14424700 -1.88200200 -1.25281800<br>O 2.70038400 -2.78141300 -0.47453300<br>H 1.81498800 -3.19475700 -0.53184700<br>O -4.85082600 -2.09056100 0.15260900<br>H -4.91631300 -2.27100500 -0.78990500<br>O 2.33890300 2.64690000 0.25120800<br>H 1.43600700 2.99029500 0.16659000<br>O -0.18809100 2.51877100 -0.55991700<br>H -1.37371800 0.20546700 -1.54093700<br>H -1.13675400 -0.85940700 1.30843700<br>C -0.02997600 2.62240000 -1.98234300<br>H 0.84392000 2.05212700 -2.30966800<br>H -0.93068300 2.26897200 -2.48800200<br>H 0.11940800 3.67935400 -2.19633300<br>H -0.32794900 1.39433400 1.20650100<br>O 0.11033300 -2.89965500 -0.41450100<br>O -2.93731200 2.20018400 -0.78126400<br>H -2.27023400 2.84318900 -0.52028100<br>O 0.61023400 0.51420700 2.80347000<br>H 1.44727100 0.99821100 2.85913100<br>O -0.31573100 1.46803600 2.45744700	1223.485350<br>Sum of electronic and thermal Enthalpies= -<br>1223.484406<br>Sum of electronic and thermal Free Energies= -<br>1223.560035
**Name**	**5- C10-H-OOH (water)**
Cartesian Coordinates	Frequency and Energy
C -3.67076300 0.79736100 -0.18031500<br>C -2.48502100 1.55940000 -0.24472600<br>C -1.23324400 0.93765900 -0.20472500<br>C -1.13926700 -0.48045500 -0.15551600<br>C -2.32200000 -1.22151400 -0.06347500<br>C -3.56257400 -0.57502700 -0.08454000<br>C -0.00942700 1.75088000 -0.12621400<br>C 0.17763300 -1.14534100 -0.13104400<br>C 1.39396700 -0.29746800 -0.46172600<br>C 1.26851300 1.05123000 0.26854700	Zero-point correction= 0.360377<br>(Hartree/Particle)<br>Thermal correction to Energy= 0.383618<br>Thermal correction to Enthalpy= 0.384562<br>Thermal correction to Gibbs Free Energy= 0.308631<br>Sum of electronic and zero-point Energies= -<br>1223.550426<br>Sum of electronic and thermal Energies= -<br>1223.527184<br>Sum of electronic and thermal Enthalpies= -

C	2.48793800	1.94564400	0.07146900	1223.526240
H	2.37423100	2.86421500	0.64921000	Sum of electronic and thermal Free Energies= -
C	3.75700300	1.22224900	0.49600500	1223.602171
C	3.90487400	-0.08306100	-0.27079700	
C	2.70586900	-0.99230000	-0.05636500	
H	-4.45721100	-1.18544800	-0.02303200	
H	3.71346900	1.01270500	1.56897300	
H	3.99476200	0.13995000	-1.34137600	
H	2.63387500	-1.25215200	1.00917800	
H	4.80973500	-0.60597400	0.04784500	
H	2.57403000	2.21973100	-0.98715100	
C	-4.99763000	1.49384400	-0.20746000	
H	-5.80984300	0.77087100	-0.14157900	
H	-5.08167000	2.19672700	0.62526000	
H	-5.11152100	2.07083700	-1.12867300	
O	-2.63483500	2.90131800	-0.32371200	
H	-1.74336200	3.30167800	-0.37585900	
O	4.90373000	2.05340300	0.32328900	
H	4.97656800	2.26617500	-0.61555600	
O	-2.34728800	-2.57864500	0.07388900	
H	-1.44887700	-2.93157700	-0.05061300	
O	0.18426700	-2.43302900	-0.71226800	
H	1.42318900	-0.11226800	-1.54557400	
H	1.15153900	0.83019200	1.34219600	
C	-0.01690400	-2.46495800	-2.13785700	
H	-0.93296600	-1.93279700	-2.40620600	
H	0.83720800	-2.02532100	-2.65510200	
H	-0.10800900	-3.51614600	-2.40400900	
H	0.29568600	-1.44004000	1.10013500	
O	-0.03456700	2.96576600	-0.31074400	
O	2.94675200	-2.17295900	-0.81714900	
H	2.18647100	-2.75833100	-0.70351000	
O	-0.99240600	-1.28435100	2.74298800	
H	-1.65510900	-1.96472900	2.53752700	
O	0.20234700	-1.81864800	2.32750100	
**Name**				**5-C10-H-OOH (pentyl ethanoate)**
Cartesian Coordinates				Frequency and Energy
C	-3.66133700	0.83562300	-0.16221800	Zero-point correction= 0.361188
C	-2.47390700	1.59949300	-0.20962000	(Hartree/Particle)
C	-1.22376200	0.97088100	-0.17801500	Thermal correction to Energy= 0.384396
C	-1.13538300	-0.44592800	-0.15355100	Thermal correction to Enthalpy= 0.385340
C	-2.31674600	-1.18772300	-0.07002700	Thermal correction to Gibbs Free Energy= 0.309535
C	-3.55630200	-0.53908700	-0.08401800	Sum of electronic and zero-point Energies= -
C	0.00006300	1.78112100	-0.06780600	1223.539348
C	0.17302200	-1.12347800	-0.16023300	Sum of electronic and thermal Energies= -
C	1.39883400	-0.27272100	-0.43664000	1223.516139
C	1.27552000	1.06339500	0.31750100	Sum of electronic and thermal Enthalpies= -
C	2.50233000	1.95203400	0.13305500	1223.515195
H	2.39461000	2.86592900	0.71870400	Sum of electronic and thermal Free Energies= -

C	3.77304100	1.21752000	0.54123700	1223.591000
C	3.90994300	-0.08382600	-0.23747300	
C	2.70410100	-0.98879700	-0.04088300	
H	-4.45178800	-1.14940700	-0.03187400	
H	3.73086500	0.99536800	1.61243600	
H	4.00605600	0.14141600	-1.30775200	
H	2.62982900	-1.25846100	1.02354700	
H	4.81036900	-0.61721000	0.07518800	
H	2.58104500	2.24492200	-0.92185400	
C	-4.98490600	1.53899700	-0.18575800	
H	-5.80359000	0.82141600	-0.13431500	
H	-5.06829600	2.23214900	0.65509900	
H	-5.09051800	2.13164400	-1.09805000	
O	-2.61295200	2.93242100	-0.26785300	
H	-1.71514600	3.32202700	-0.30041400	
O	4.91999500	2.04102300	0.38302100	
H	5.00454300	2.25807700	-0.55236200	
O	-2.32710200	-2.54054000	0.08026000	
H	-1.44689000	-2.88710900	-0.14783700	
O	0.16105300	-2.37199600	-0.82857800	
H	1.44558000	-0.05936400	-1.51566100	
H	1.15817400	0.82930000	1.38838200	
C	0.02384400	-2.30397600	-2.25809100	
H	-0.82161400	-1.66787100	-2.53448700	
H	0.94211400	-1.92958100	-2.71429800	
H	-0.15866500	-3.32383300	-2.59376200	
H	0.26053600	-1.54870500	1.06474400	
O	-0.02171100	2.99757800	-0.21468100	
O	2.93540100	-2.15282800	-0.81830600	
H	2.18418100	-2.74511700	-0.69832700	
O	-1.05548100	-1.58606800	2.66491900	
H	-1.76785200	-2.14401000	2.31073100	
O	0.09158800	-2.14368400	2.15339300	
**Name**				**Trolox-H-OOH** (gas)
Cartesian Coordinates				Frequency and Energy
C	-1.91851700	0.94223300	-0.63651500	Zero-point correction= 0.313503
C	-0.61618900	1.42081500	-0.60769000	(Hartree/Particle)
C	0.43886500	0.49222400	-0.59978000	Thermal correction to Energy= 0.334403
C	0.23235200	-0.89550000	-0.62778300	Thermal correction to Enthalpy= 0.335347
C	-1.07279800	-1.37726300	-0.61217100	Thermal correction to Gibbs Free Energy= 0.263330
C	-2.15246300	-0.46104100	-0.62350900	Sum of electronic and zero-point Energies= -
C	1.40343800	-1.84946300	-0.67742500	995.726218
C	2.69321600	-1.12306200	-1.03773600	Sum of electronic and thermal Energies= -995.705318
C	2.80167800	0.17249500	-0.24996700	Sum of electronic and thermal Enthalpies= -
H	1.52143100	-2.34694300	0.29161300	995.704374
H	3.56326000	-1.74382000	-0.82383500	Sum of electronic and thermal Free Energies= -
C	-1.34820200	-2.85420900	-0.61270500	995.776391
H	-1.13447200	-3.28770800	-1.59515700	
H	-0.71813100	-3.37149400	0.11436800	

H -2.39423000 -3.04630800 -0.38542000<br>C -0.34075400 2.90303600 -0.57205500<br>H -0.60580500 3.36893500 -1.52540700<br>H -0.93871000 3.38597400 0.20370300<br>H 0.71053700 3.11203800 -0.38742100<br>C -3.09448000 1.87436300 -0.63416000<br>H -2.88534600 2.77860800 -1.20623200<br>H -3.97279400 1.38635900 -1.05348400<br>H -3.33736100 2.16908500 0.39231900<br>C 4.05772000 0.96244400 -0.59329400<br>H 4.07929600 1.90861400 -0.04920600<br>H 4.94055400 0.38005600 -0.32775000<br>H 4.06788400 1.17266700 -1.66371500<br>H 2.70576900 -0.85937200 -2.09920200<br>H 1.20068100 -2.63658900 -1.40718100<br>C 2.82226700 -0.11648700 1.26579600<br>O 2.17324600 0.76711600 2.03408800<br>H 1.73152300 1.41609700 1.46859500<br>O -3.39017300 -0.92577500 -0.64978000<br>H -3.90297900 -0.64973900 0.27091300<br>O 1.70094300 1.03571300 -0.55182800<br>O 3.42560100 -1.03764100 1.73050700<br>O -2.95467800 0.13925300 1.94416800<br>O -4.14606600 -0.36579400 1.52501400<br>H -2.53889500 -0.58295300 2.43911100	
**Name**	**Trolox-H-OOH** (pentyl ethanoate)
Cartesian Coordinates	Frequency and Energy
C -1.90822200 0.98387200 -0.61749300<br>C -0.59817800 1.44105000 -0.56881400<br>C 0.44234100 0.49430500 -0.57703800<br>C 0.21353900 -0.88988700 -0.61842700<br>C -1.10022500 -1.35034700 -0.61632000<br>C -2.16286400 -0.41505400 -0.63296800<br>C 1.37087300 -1.85958000 -0.66882700<br>C 2.66689400 -1.15325400 -1.04401700<br>C 2.81088100 0.14240800 -0.26344100<br>H 1.48129300 -2.35392700 0.30296000<br>H 3.53067300 -1.78821700 -0.84698700<br>C -1.39488200 -2.82273100 -0.62659300<br>H -1.21955000 -3.24745800 -1.62092300<br>H -0.74770200 -3.35587600 0.07331200<br>H -2.43412900 -3.01086900 -0.36445600<br>C -0.29937500 2.91640300 -0.50300900<br>H -0.47133900 3.39206600 -1.47371300<br>H -0.95078600 3.40821800 0.22212200<br>H 0.73524000 3.10754900 -0.22454100<br>C -3.06251600 1.94265800 -0.61568900<br>H -2.84394300 2.82238700 -1.22320600<br>H -3.96477700 1.46980300 -1.00038800	Zero-point correction= 0.312677 (Hartree/Particle)<br>Thermal correction to Energy= 0.333797<br>Thermal correction to Enthalpy= 0.334741<br>Thermal correction to Gibbs Free Energy= 0.261634<br>Sum of electronic and zero-point Energies= -995.753273<br>Sum of electronic and thermal Energies= -995.732154<br>Sum of electronic and thermal Enthalpies= -995.731210<br>Sum of electronic and thermal Free Energies= -995.804317

H -3.27212100 2.28562400 0.40320300<br>C 4.06466600 0.91912900 -0.64183100<br>H 4.11765900 1.86378000 -0.09606300<br>H 4.95084900 0.32630900 -0.41126300<br>H 4.04289300 1.13090300 -1.71216800<br>H 2.66956500 -0.89118200 -2.10585300<br>H 1.15498100 -2.64787100 -1.39315200<br>C 2.86486500 -0.12835600 1.25277200<br>O 2.24356900 0.76641400 2.02797700<br>H 1.80657100 1.42880700 1.47109500<br>O -3.41201600 -0.86143700 -0.70359300<br>H -3.92588400 -0.63818500 0.21012100<br>O 1.70963700 1.01846500 -0.53671600<br>O 3.47184500 -1.04651500 1.72633000<br>O -3.00889300 0.05553700 1.96048900<br>O -4.19598400 -0.42245900 1.50816300<br>H -2.57154400 -0.70559600 2.37888500	
**Name**	**Trolox-H-OOH (**water) (anion)
Cartesian Coordinates	Frequency and Energy
C -1.85551000 1.34558000 -0.31642900<br>C -0.51294600 1.68768100 -0.22354400<br>C 0.46225200 0.69143700 -0.43703000<br>C 0.13742900 -0.63633900 -0.76026900<br>C -1.20811400 -0.98241800 -0.84246700<br>C -2.19466800 0.01151000 -0.64894900<br>C 1.23679000 -1.63990600 -1.02580600<br>C 2.56529900 -0.93528900 -1.27261200<br>C 2.79134300 0.13443200 -0.21141300<br>H 1.32849300 -2.32183900 -0.17201200<br>H 3.39208400 -1.64528900 -1.25647300<br>C -1.65561600 -2.38882500 -1.12667600<br>H -0.85948400 -3.10892900 -0.94732400<br>H -2.50662100 -2.65027600 -0.49273600<br>H -1.98540300 -2.49234700 -2.16529700<br>C -0.04518200 3.07499500 0.12172900<br>H -0.87551500 3.77371200 0.19576400<br>H 0.49230800 3.07318000 1.07450200<br>H 0.65002500 3.44707500 -0.63459700<br>C -2.94415700 2.34873700 -0.04828600<br>H -2.79669600 2.82488500 0.92356900<br>H -2.94454800 3.14068700 -0.80240200<br>H -3.92204400 1.87267400 -0.05343800<br>C 4.08625700 0.89958800 -0.42679700<br>H 4.19863900 1.68126700 0.32816700<br>H 4.93402400 0.21798700 -0.35778900<br>H 4.08101800 1.35992700 -1.41713800<br>H 2.55990400 -0.43948600 -2.24820000<br>H 0.97421200 -2.25294700 -1.88995400<br>C 2.78488500 -0.49687800 1.20896200	Zero-point correction= 0.299250 (Hartree/Particle)<br>Thermal correction to Energy= 0.320113<br>Thermal correction to Enthalpy= 0.321057<br>Thermal correction to Gibbs Free Energy= 0.248581<br>Sum of electronic and zero-point Energies= -995.318391<br>Sum of electronic and thermal Energies= -995.297528<br>Sum of electronic and thermal Enthalpies= -995.296584<br>Sum of electronic and thermal Free Energies= -995.369060

O	1.94185800	-0.09150400	2.04074700	
O	-3.49122500	-0.32248100	-0.83351300	
H	-3.84960900	-0.78552800	0.02192600	
O	1.74590800	1.11753800	-0.31565000	
O	3.65638700	-1.37956900	1.40744100	
O	-2.73783900	-0.89630500	1.85347800	
O	-3.94239400	-1.26869900	1.37830400	
H	-2.14072800	-1.65241600	1.70451300	

References

(1) Rimarčík, J.; Lukeš, V.; Klein, E.; Ilčin, M. Study of the Solvent Effect on the Enthalpies of Homolytic and Heterolytic N–H Bond Cleavage in P-Phenylenediamine and Tetracyano-P-Phenylenediamine. *J. Mol. Struct: THEOCHEM* **2010,** *952*, 25-30.

(2) Wright, J. S.; Johnson, E. R.; DiLabio, G. A. Predicting the Activity of Phenolic Antioxidants: Theoretical Method, Analysis of Substituent Effects, and Application to Major Families of Antioxidants. *J. Am. Chem. Soc.* **2001,** *123*, 1173-1183.

(3) Alvarez-Idaboy, J. R. l.; Galano, A. On the Chemical Repair of DNA Radicals by Glutathione: Hydrogen Vs Electron Transfer. *J. Phys. Chem. B* **2012,** *116*, 9316-9325.

(4) Galano, A.; Alvarez-Idaboy, J. R. Kinetics of Radical-Molecule Reactions in Aqueous Solution: A Benchmark Study of the Performance of Density Functional Methods. *J. Comput. Chem.* **2014,** *35*, 2019-2026.

(5) Galano, A.; Raúl Alvarez-Idaboy, J. Computational Strategies for Predicting Free Radical Scavengers' Protection against Oxidative Stress: Where Are We and What Might Follow? *Int. J. Quantum Chem.* **2019,** *119*, e25665.

(6) Thong, N. M.; Duong, T.; Pham, L. T.; Nam, P. C. Theoretical Investigation on the Bond Dissociation Enthalpies of Phenolic Compounds Extracted from Artocarpus Altilis Using Oniom (Rob3lyp/6-311++ G (2df, 2p): Pm6) Method. *Chem. Phys. Lett.* **2014,** *613*, 139-145.

(7) Thong, N. M.; Quang, D. T.; Bui, N. H. T.; Dao, D. Q.; Nam, P. C. Antioxidant Properties of Xanthones Extracted from the Pericarp of Garcinia Mangostana (Mangosteen): A Theoretical Study. *Chem. Phys. Lett.* **2015,** *625*, 30-35.

(8) Bartmess, J. E. Thermodynamics of the Electron and the Proton. *J. Phys. Chem.* **1994,** *98*, 6420-6424.

(9) Urbaniak, A.; Szeląg, M.; Molski, M. Theoretical Investigation of Stereochemistry and Solvent Influence on Antioxidant Activity of Ferulic Acid. *Comput. Theor. Chem.* **2013,** *1012*, 33-40.

(10) Donald, W. A.; Demireva, M.; Leib, R. D.; Aiken, M. J.; Williams, E. R. Electron Hydration and Ion–Electron Pairs in Water Clusters Containing Trivalent Metal Ions. *J. Am. Chem. Soc.* **2010,** *132*, 4633-4640.
